# Microbiome analysis of the restricted bacteria in radioactive element-containing water at the Fukushima Daiichi Nuclear Power Station

**DOI:** 10.1128/aem.02113-23

**Published:** 2024-03-12

**Authors:** Tomoro Warashina, Asako Sato, Hiroshi Hinai, Nurislam Shaikhutdinov, Elena Shagimardanova, Hiroshi Mori, Satoshi Tamaki, Motofumi Saito, Yukihisa Sanada, Yoshito Sasaki, Kozue Shimada, Yuma Dotsuta, Toru Kitagaki, Shigenori Maruyama, Oleg Gusev, Issay Narumi, Ken Kurokawa, Teppei Morita, Toshikazu Ebisuzaki, Akihiko Nishimura, Yoshikazu Koma, Akio Kanai

**Affiliations:** 1 Institute for Advanced Biosciences, Keio University, Tsuruoka, Japan; 2 Systems Biology Program, Graduate School of Media and Governance, Keio University, Fujisawa, Japan; 3 Japan Atomic Energy Agency, Tokai, Japan; 4 Regulatory Genomics Research Center, Institute of Fundamental Medicine and Biology, Kazan (Volga Region) Federal University, Kazan, Russia; 5 Life Improvement by Future Technologies (LIFT) Center, Skolkovo, Moscow, Russia; 6 Loginov Moscow Clinical Scientific Center, Moscow, Russia; 7 National Institute of Genetics, Mishima, Japan; 8 Earth-Life Science Institute, Tokyo Institute of Technology, Tokyo, Japan; 9 Intractable Disease Research Center, School of Medicine, Juntendo University, Tokyo, Japan; 10 Faculty of Life Sciences, Toyo University, Oura-gun, Japan; 11 Computational Astrophysics Laboratory, RIKEN, Wako, Japan; 12 Faculty of Environment and Information Studies, Keio University, Fujisawa, Japan; Kyoto University, Kyoto, Japan

**Keywords:** 16S amplicon sequencing, torus room water, Fukushima Daiichi NPS, radiation resistance

## Abstract

A major incident occurred at the Fukushima Daiichi Nuclear Power Station following the tsunami triggered by the Tohoku–Pacific Ocean Earthquake in March 2011, whereby seawater entered the torus room in the basement of the reactor building. Here, we identify and analyze the bacterial communities in the torus room water and several environmental samples. Samples of the torus room water (1 × 10^9^ Bq^137^Cs/L) were collected by the Tokyo Electric Power Company Holdings from two sampling points between 30 cm and 1 m from the bottom of the room (TW1) and the bottom layer (TW2). A structural analysis of the bacterial communities based on 16S rRNA amplicon sequencing revealed that the predominant bacterial genera in TW1 and TW2 were similar. TW1 primarily contained the genus *Limnobacter*, a thiosulfate-oxidizing bacterium. γ-Irradiation tests on *Limnobacter thiooxidans*, the most closely related phylogenetically found in TW1, indicated that its radiation resistance was similar to ordinary bacteria. TW2 predominantly contained the genus *Brevirhabdus*, a manganese-oxidizing bacterium. Although bacterial diversity in the torus room water was lower than seawater near Fukushima, ~70% of identified genera were associated with metal corrosion. Latent environment allocation—an analytical technique that estimates habitat distributions and co-detection analyses—revealed that the microbial communities in the torus room water originated from a distinct blend of natural marine microbial and artificial bacterial communities typical of biofilms, sludge, and wastewater. Understanding the specific bacteria linked to metal corrosion in damaged plants is important for advancing decommissioning efforts.

**IMPORTANCE:**

In the context of nuclear power station decommissioning, the proliferation of microorganisms within the reactor and piping systems constitutes a formidable challenge. Therefore, the identification of microbial communities in such environments is of paramount importance. In the aftermath of the Fukushima Daiichi Nuclear Power Station accident, microbial community analysis was conducted on environmental samples collected mainly outside the site. However, analyses using samples from on-site areas, including adjacent soil and seawater, were not performed. This study represents the first comprehensive analysis of microbial communities, utilizing meta 16S amplicon sequencing, with a focus on environmental samples collected from the radioactive element-containing water in the torus room, including the surrounding environments. Some of the identified microbial genera are shared with those previously identified in spent nuclear fuel pools in countries such as France and Brazil. Moreover, our discussion in this paper elucidates the correlation of many of these bacteria with metal corrosion.

## INTRODUCTION

**Fig 1:**
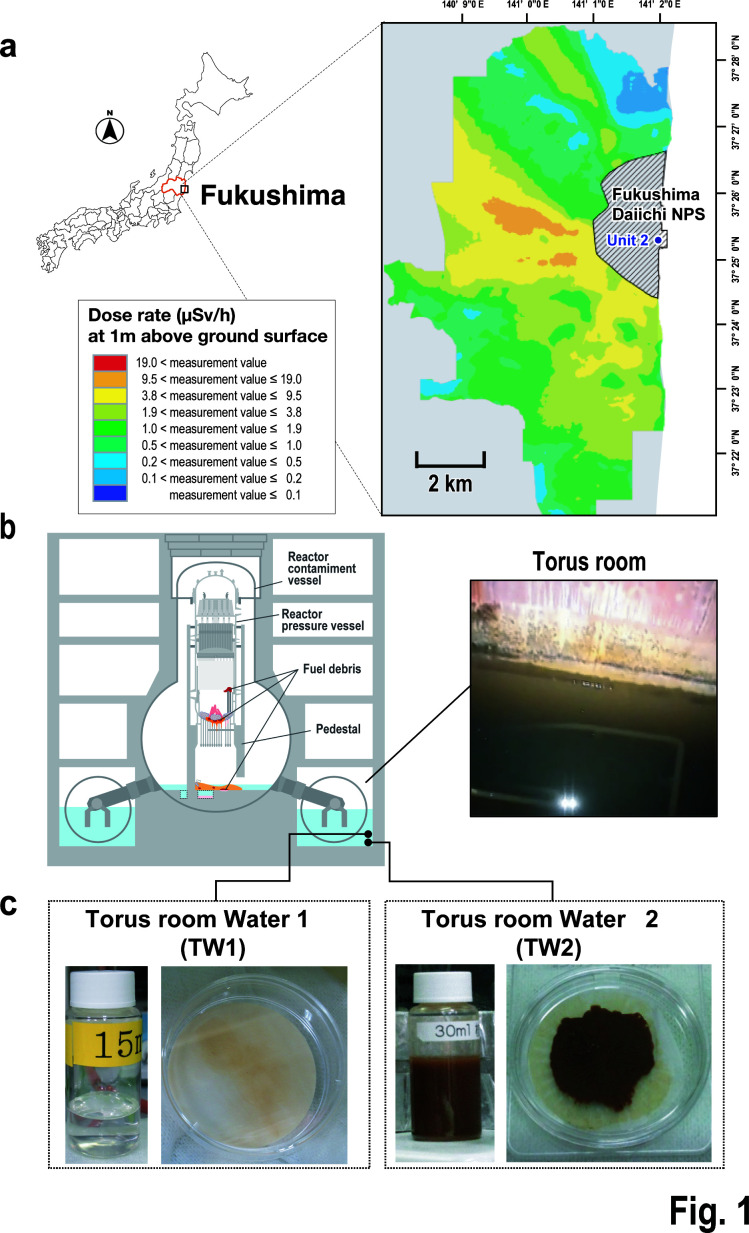
Summary of waters obtained from the torus room of the unit 2 reactor at the Fukushima Daiichi Nuclear Power Station (NPS). (**a**) Location of Fukushima Prefecture (surrounded by a red line) in Japan and the location of Fukushima Daiichi NPS on an enlarged map of Fukushima Prefecture. Results of a radiation survey conducted by the Regulatory Agency in 2019 by a manned helicopter and an unmanned helicopter (JAEA, Database for Radioactive Substance Monitoring Data, https://emdb.jaea.go.jp/emdb/top) were used as the background figure. Background map is copyrighted by ESRI Japan. (**b**) Cross-section of the unit 2 reactor building and a photograph of the torus room waters (provided by the Tokyo Electric Power Company Holdings, Inc). (**c**) Photographs of each vial containing torus room water 1 (TW1) or 2 (TW2) and of each filter (0.45-µm pore size) used to filter the water samples. TW1 was collected from the upper and middle parts of the torus room, and TW2 was collected from the lowest part, which contained abundant residue (brown color).

The Fukushima Daiichi Nuclear Power Station (NPS) located in Fukushima Prefecture, Japan, experienced an influx of seawater into units 1–4 during the tsunami triggered by an earthquake in March 2011. At present, these buildings are believed to retain the seawater that flowed in during the disaster (1). The torus room, which is situated beneath the reactor building (Fig. 1a; Fig. S1), houses a pool designed to trap high-pressure steam containing radioactive materials when the pressure inside the primary containment vessel rises. The stagnant water was sampled from the torus room of the unit 2 and radiochemically analyzed to manage the water (2). It is postulated that microbiome originated from the seawater had been exposed to radiation in the radioactive water, and information of microorganisms is important to understand the environment of the stagnant water for sustainable decommissioning work. It is well established that ionizing radiation emitted from nuclear fuel can directly damage DNA. Moreover, when such ionizing radiation interacts with intracellular water molecules, radicals are generated that can also damage DNA and other molecular constituents of the cell. Conversely, certain microorganisms are known to have mechanisms that confer resistance to high levels of ionizing radiation (3). This raises the possibility that radiation-resistant bacteria inhabit the interiors of nuclear reactors.

In the history of nuclear accidents, several incidents associated with nuclear power plants have been on a scale similar to or larger than the Fukushima Daiichi NPS accident, and reports of the microbial communities in radioactively contaminated environments have been published. For instance, following the core meltdown incident at the Three Mile Island nuclear power plant in the United States in 1975, a proliferation of algae was seen in the illuminated contaminated water during decommissioning operations (4, 5). Biofilm formation within the water treatment piping has also been reported (6). After the 1986 accident at the Chernobyl Nuclear Power Station in the former Soviet Union, a metagenomic 16S analysis of the soil was conducted and showed a reduction in bacterial diversity under radioactive contamination conditions (7). Taxa displaying strong resistance to reactive oxygen species were also identified (8). Other reports have suggested the predominance of species containing melanin pigments in heavily contaminated areas inside the containment vessel (9).

Several reports have examined the impact of the release of radioactive substances into the environment of Fukushima Prefecture after the Fukushima Daiichi NPS accident (10, 11). In 2021, Ihara et al. conducted a metagenomic analysis of soils with varying contamination levels and reported that although there were no significant changes in the major microbial communities in response to radioactive contamination, the proportion of radiation-resistant bacteria, such as *Geodermatophilus bullaregiensis*, which exists at <1% in low-dose (20,000 Bq^137^Cs/kg) contaminated soils, was approximately 0.1% higher in soils with higher radioactivity levels (563,000 Bq^137^Cs/kg). In 2019, Hernandez et al. compared the microbial community structures in soil samples from areas with low radioactivity (860 Bq^137^Cs/kg) and high radioactivity (34,430 Bq^137^Cs/kg) and found similarities in the major microbial communities in the two areas (10). Previous studies have been based primarily on samples collected outside the Fukushima Daiichi NPS, and there have been no reports of analyses of the microbial communities within the site or inside the reactor buildings. However, as previously mentioned, within the Fukushima Daiichi NPS reactor, objects resembling biofilms have been observed on the surfaces of metals. Therefore, it is essential to analyze the microbial communities and their characteristics within the Fukushima Daiichi NPS, including those inside the reactor. Some microorganisms are known to form biofilms on metal surfaces, which can promote metal corrosion (12). Given the expected prolongation of the decommissioning process, understanding the microorganisms associated with metal corrosion in the reactor buildings and the residual water is considered extremely important for inhibiting corrosion in the reactor and buildings.

Therefore, we comprehensively analyzed these microbial community structures based on 16S rRNA sequences, focusing on the water in the torus room, which has been retained within the reactor containment vessel for approximately 9 years. Because the samples contained radioactive substances, it was essential to conduct the analysis in the radiation control area of an authorized nuclear facility, with measures to prevent the release of radioactive materials into the external environment. Large sequencers are commonly used in microbial community analyses, such those of Illumina (13). However, because the available space and utilities were limited within the radiation control area, these experiments were conducted using a small PCR device and a portable MinION sequencer (Oxford Nanopore Technologies, Oxford, UK). The results revealed a limited microbial presence in the torus room water, which predominantly contained a mixture of bacteria detected in biofilms and sludge and those found in marine environments. Approximately 70% of the genera comprising the microbial communities within the torus room were related to bacteria known to be associated with metal corrosion.

## MATERIALS AND METHODS

### Environmental samples

#### Samples inside the nuclear reactor at the Fukushima Daiichi NPS

The Fukushima Daiichi NPS is located in Futaba-gun, Fukushima Prefecture, Japan (Fig. 1a). The torus room water samples (Fig. 1b; Table S1) were collected twice by the Tokyo Electric Power Company Holdings, Inc., from the torus room of the unit 2 reactor of the Fukushima Daiichi NPS (37°25ʹ20.1ʺN 141°01ʹ57.7ʺE). Water in the torus room reached a height of ~4.6 m. The radioactive water was transferred and decontaminated to recover freshwater, and the water under the reactor building had been reduced to a height of ~1 m when the samples were collected. The volume of the torus room water at this point was ~1,900 m^3^, with radioactivity of 2.8 × 10^14^ Bq. Torus room water sample 1 (TW1) was a mixture of samples collected by remote pumping on 13 February 2020 at 0.3 and 1 m from the bottom of the torus room. Torus room water sample 2 (TW2) was dredged on 30 June 2020, from the lowest level of the torus room, in which a reddish-brown residue had been deposited. TW2 was collected using dedicated water intake equipment. Samples were stored in sealed vials. During analysis, all equipment used was sterilized using ethanol, and operations were conducted on a clean bench to minimize the risk of contamination. Physicochemical information such as the radionuclides, pH, and ion concentrations of the torus room water samples is summarized in Table S2. The torus room water samples were sealed in vials and stored at room temperature during the period from collection to DNA extraction (Fig. 1c).

#### Samples from environments near Fukushima Daiichi NPS and other environments

In addition to the torus room water samples, environmental samples from soil (SO1–SO6), seawater (SW1–SW5), seabed soil (SS1 and SS2), and freshwater (RW1 and RW2) were collected. The sampling data and physicochemical information on these samples are also summarized in Table S1. Soil samples were collected from a forest approximately 1 km southwest of the unit 2 reactor at the Fukushima Daiichi NPS. The radiation in this soil area, measured with an ionization chamber, was 71.0 µSv/h at 5 cm and 48.2 µSv/h at 100 cm from the ground surface. Three samples (SO1–SO3) were collected from the soil surface layer close to each other (within 200 cm). Three soil samples were collected at different depths from the ground surface: 0–2 cm (SO4), 6–7 cm (SO5), and 11–13 cm (SO6). As the control samples for the soil in Fukushima Prefecture, we used the soil microbial community structure data (10) (YC and MC samples) from the Yamakiya district (town of Kawamata), approximately 40 km from the Fukushima Daiichi NPS (Fig. S1a). The samples of seawater, seabed soil, and the seawater on the seabed soil off the coast of Fukushima Prefecture were collected by a Japan Atomic Energy Agency (JAEA) research vessel. As shown in Fig. S1b, the seabed soil (SS1) and the seawater on the seabed soil (SW1) were collected off the Odaka River, and the seabed soil (SS2) and the seawater on the seabed soil (SW2) were collected off the Ukedo River in Fukushima Prefecture. Surface seawater (SW3) was sampled off the coast of the Fukushima Daiichi NPS. As control samples, surface seawater samples were taken off the coast at the Hitachi Port (SW4) and at the Hitachi Port (SW5), Ibaraki Prefecture, Japan. Freshwater samples (RW1 and RW2) were collected twice on different days from the running water of the Yashiro River flowing through the town of Ishikawa, Fukushima Prefecture (Fig. S1c). The Microbial Community Standard D6300 (Zymo Research, Irvine, CA, USA) was used as the defined microbial input in our series of experiments.

### Environmental DNA preparation and 16S rRNA amplicon sequencing

#### Preparation of environmental DNAs and 16S rRNA amplicon sequencing with the MinION platform

TW1 was a sample of 30 mL, containing a mixture of 15 mL of torus room water samples collected either from approximately 0.3 or 1 m above the bottom of the torus room. TW1 was filtered through a sterile 0.22-µm membrane filter to trap the microorganisms. The filter was washed with 30 mL of 1× phosphate-buffered saline (Thermo Fisher Scientific, MA, USA) to reduce the amount of radioactivity in the filtered sample. The bacterial genomic DNA was extracted from the filter using the DNeasy PowerWater Kit (Qiagen, Venlo, Netherlands) according to the protocol specified by the manufacturer. A DNA library of 16S rRNA amplicons for sequencing on the MinION sequencer (Oxford Nanopore Technologies, Oxford, UK） was constructed with PCR using the 27F/1492R primer set (Table S3) and the 16S Barcoding Kit (SQK-RAB204; Oxford Nanopore Technologies, Oxford, UK), based on the manufacturer’s protocol. However, because the amount of genomic DNA obtained was extremely small, the PCR was performed at template concentration below the recommended concentration, and the number of PCR cycles was increased from 25 to 30. The DNA library was then sequenced using the MinION sequencer with a Flow Cell (FLO-MINI106; Oxford Nanopore Technologies). The TW2 sample was a 15-mL sample taken from the bottom layer of the torus room, and the microorganisms were trapped on a membrane filter in the same manner as for TW1. A 16S amplicon sequencing approach using molecular barcodes was used to process the genomic DNA obtained from TW2. A DNA library of 16S rRNA amplicons was constructed with the Ligation Sequencing Kit (SQK-LSK109; Oxford Nanopore Technologies), and the library was then sequenced with the MinION sequencer using a Flow Cell (FLO-MINI111; Oxford Nanopore Technologies). These experiments were performed with the reagents and protocol reported by Karst et al. However, the number of PCR cycles was increased from 25 to 30, and the DNA polymerase used was KOD FX Neo (Toyobo, Osaka, Japan), which has good amplification efficiency in the presence of many contaminants.

For the aqueous environmental samples other than the torus room samples, the microorganisms were trapped on a 0.22-µm membrane filter, and the genomic DNA was extracted from the filter using the DNeasy PowerWater Kit (Qiagen), according to the manufacturer’s protocol. Genomic DNA was extracted from the soil samples using the ISOIL kit (Nippon Gene Co., Ltd, Tokyo, Japan), according to the protocol recommended by the manufacturer. A DNA library of 16S amplicons was constructed and sequenced as for TW1. In this study, the MinION sequencer was controlled by the MinIT device (Oxford Nanopore Technologies).

#### 16S rRNA amplicon sequencing with the Illumina platform

In this analysis, we used bacterial universal primers (341f and 785r) for PCR, which amplify the V3–V4 region of 16S rRNA, according to a previous report (14). The PCR was performed using the KOD FX Neo polymerase (Toyobo), according to the manufacturer’s protocol. In the PCR, initial denaturation was performed at 94°C for 2 min, followed by 35 cycles of 98°C for 10 s, 60°C for 30 s, and 68°C for 30 s. The resulting PCR product was separated using 1.5% agarose gel electrophoresis and then excised and purified. Library construction and the sequencing of the amplicons were outsourced to GeneBay Co., Ltd. (Yokohama, Japan), and performed with paired-end reads (250 bases × 2) using the Illumina NovaSeq 6000 (San Diego, CA, USA). The PCR primers used in this study are summarized in Table S3.

### Analysis of nucleotide sequence data and microbial community structures

#### For the MinION sequencer

To obtain nucleotide sequences (reads) from the MinION sequencer, base calling and barcode sorting (i.e., demultiplexing) were performed using the MinKnow software (Oxford Nanopore Technologies) on the MinIT device. For TW2, the fast5 file output from MinIT was base-called again with Guppy (v5.0.17). The fastq files obtained with base calling were subjected to quality control (QC) with the following procedure (see Fig. S2). First, we used Porechop (v0.2.4; https://github.com/rrwick/Porechop) to remove the adapter sequences. The adapter sequences at the ends of the read were trimmed, and if there was an adapter sequence in the middle of the read, the sequence was treated as a chimera and removed. Reads with a Q-score of ≤10 (10% error rate) and reads shorter than 1.2 kb or longer than 1.8 kb were removed using Nanofilt (v0.2.4) (15) because the estimated 16S rRNA length is approximately 1.5 kb. The sequences were then analyzed using NanoStat (v1.1.2) (15) to examine the quality and length distribution of the reads. To estimate the microbial community structures, we used the obtained reads as the query sequences and performed a sequence similarity search (E-value cutoff value of 1e^−5^) using the BLASTN program (version: 2.9.0) (16) against the 16S/18S ribosomal RNA database of SILVA 138 SSU (17). In this analysis, microorganisms with a sequence identity of ≥90% and with the highest matching score between the query sequence and the 16S rRNA sequence registered in the database were defined as microorganisms using the query sequence.

#### For the Illumina sequencer

The sequence reads (250 bases × 2) prepared by GeneBay Co., Ltd., contained adapter sequences and low-quality reads, so QC was performed with the following procedure. First, reads containing adapter sequences and low-quality reads (average sequencing error rate >1%) were removed using cutadapt v3.2 (18). QIIME2 (v2019.10) (19), a metagenomic analysis pipeline tool, was then used to trim the primer sequences at the beginning of the reads (5′ region) and low-quality sequences at the ends of the reads (3′ region) and to merge the reads to obtain the entire amplicon sequences. Noise was removed using the DADA2 model (20), which corrects sequencing errors in QIIME2, and representative reads were selected. To determine to which microorganisms the obtained read belonged, a sequence similarity search (E-value cutoff value of 1e^−5^) was performed against the 16S/18S ribosomal RNA database of SILVA 138 SSU (17) with the BLASTN program (v2.9.0) (16), using the representative read sequence as the query sequence. In this analysis, microorganisms with a sequence identity of ≥97% and with the highest matching score between the query sequence and the 16S rRNA sequence registered in the database were defined as microorganisms using the query sequence. A hierarchical cluster analysis was performed using Ward’s method with Scipy (v1.5.0) (21).

### Latent environment allocation

We used latent environment allocation (LEA) (22) to analyze the environmental topics to which the bacteria identified belonged. In LEA, the microbial community structure and metadata registered in the metagenomic database MicrobeDB.jp were analyzed with reference to a set 80 environmental topics. To perform the LEA analysis, the microbial community structure was estimated again with VITCOMIC2 (23). The microbial community structure data were then visualized on a two-dimensional map with LEA Global Map (http://leamicrobe.jp/).

### Analysis of microbial diversity in the environments

To compare the diversity of the microbial communities, we calculated the number of genera and the Shannon–Wiener diversity index (H) as follows:


$$H=-\sum_{i=1}^{s}\left(p_{i}{log}_{2}p_{i}\right)$$

where S is the number of genera and $p_{i}$ is the proportion of the community represented by genera.

### γ-Irradiation of *L. thiooxidans*

*L. thiooxidans* CS-K2 was obtained from Deutsche Sammlung von Mikroorganismen und Zellkulturen (Braunschweig, Germany) and was cultured in Bacto Marine Broth (Difco 2216) with shaking at 30°C for 24 h. The bacterial cultures were then subjected to γ-irradiation at 0, 2.5, and 5 Gy/h for 6 days at the National Institutes for Quantum Science and Technology, Takasaki, Japan. After irradiation, serial dilutions (10^−1^–10^−7^) of the culture medium were prepared and cultured on *Limnobacter* medium plates at 30°C for 48 h. The number of colonies was counted.

### Analysis of co-detection rates at the genus level using MicrobeDB.jp

MicrobeDB.jp (22) is a database containing approximately 60,000 microbial community structure data entries. The data registered in the database were acquired in January 2023 from the Life Science Database Archive of the Japan Science and Technology Agency (https://dbarchive.biosciencedbc.jp/en/microbedb/download.html). Initially, metagenomic data containing specific bacterial genera (as displayed in the columns of the heatmap) were extracted from the entirety of MicrobeDB.jp. The co-detection rate was defined as the proportion of extracted metagenomic data containing particular bacterial genera (as represented in the rows of the heatmap). The co-detection rates were depicted in a heatmap. Both the computation of the co-detection rates and their visualization in the heatmap were performed using Biopython (24).

### Statistical analysis

Statistical analyses were performed using Biopython (24). Data were expressed as mean ± SD or plots. The Student’s *t* test was used to compare two groups.

## RESULTS AND DISCUSSION

### Torus room water samples from the unit 2 reactor of Fukushima Daiichi NPS

The water examined in this analysis had remained in the torus room in the basement of the unit 2 reactor building, which houses the suppression chamber (Fig. 1b), for approximately 9 years in a semi-enclosed environment until it was sampled in 2020. When observed in the vials, TW1 was a transparent liquid, whereas TW2 contained a large amount of reddish-brown residue that appeared to be sediment (Fig. 1c). Table S2 shows the physicochemical properties of the torus room water and the ion concentrations of the filtrates and residues. In brief, both TW1 and TW2 were neutral solutions (around pH 7.5) with redox potentials of ~400 mV, and both contained approximately 1 × 10^9^ Bq ^137^Cs/L (Table S2a). The concentrations of elements such as Na, Mg, K, Ca, and Sr in TW1 and TW2 (Table S2b) were close to those of seawater (25). The chloride ion concentrations in TW1 and TW2 differed slightly, with 14,000 ppm in TW1 and 20,000 ppm in TW2. Because the chloride ion concentration of seawater is usually around 20,000 ppm (25), this suggests that the water sampled from the torus chamber comprised mainly seawater. TW2 was closer to seawater compared with TW1. The torus room water samples were filtered using 0.22-µm sterilized filters to trap the microorganisms. A thin reddish-brown residue was observed on the filter of TW1, and a reddish-brown solid-content-rich residue was observed on that of TW2 (Fig. 1c). Elemental analyses of these residues showed that about 90% was Na in TW1, whereas almost no Na was detected in the TW2 residue. However, approximately 80% of the TW2 residue was composed of Fe, which was detected negligibly in the TW1 residue (Table S2b). Based on these physicochemical measurements, it can be inferred that the seawater that entered the reactor building owing to the tsunami was retained in the bottom layer (TW2) of the torus room water and that the upper layer (TW1) contained seawater diluted by freshwater. Therefore, samples TW1 and TW2 represent the microbial communities at different depths.

### Microbial community structure of torus room water samples

Environmental DNA was extracted from the microbes trapped on the 0.22-µm filters, and 30 mL of TW1 yielded 9 ng of DNA (0.3 ng per 1 mL) and 15 mL of TW2 yielded 4 ng of DNA (0.27 ng per 1 mL) (Table S3). Using these DNAs as templates, each 16S rRNA gene region was amplified, and the nucleotide sequence was determined using a MinION sequencer. We obtained 2,673k reads for TW1 and 368k reads for TW2. Because the reads obtained included fragmented sequences and low-quality reads, QC was undertaken based on read length and Q-score (Fig. S2). After QC, the numbers of sequences available for accurate analysis were 1,027k reads for TW1 and 16.5k reads for TW2.

To assign the 16S rRNA sequences obtained to the appropriate microbiota, it was necessary to appropriately set the BLASTN threshold for nucleotide similarity analysis. Therefore, we determined the nucleotide sequence of 16S rRNAs with a mock bacterial community using the same method as that used for TW1. A mock bacterial community is a set of eight known bacterial genera that is used as the positive control. As shown in Fig. S3, if the alignment length is 1,000–1,500 bp and the percentage identity is ≥95%, an organism is classified into the correct community, at least at the genus level. Below this threshold, some reads would be misclassified, and the number of genera will be evaluated higher than the actual microbial community. Therefore, we estimated the numbers of genera in the samples (diversity analysis) by setting a threshold of at least 95% identity with an alignment length of at least 1,000 bp. However, only approximately 0.4% of the reads exceeded the threshold defined above, since more reads are needed to reflect the entire microbial community structure. To estimate the community structure, we used a sequence identity threshold of 90% for bacterial genus identification, as is commonly used in study (26) of 16S rRNA sequences obtained using the Nanopore technology. A total of 63.0% of the reads passed this threshold for TW1, and 92.6%, for TW2. The alignment length used was 1,000 bp, consistent with the diversity analysis previously mentioned (Fig. S3). When we analyzed the torus room water data using this threshold, we confirmed that the ranking of the five most abundant bacterial species (accounting for ~90% of the microbial communities) did not change according to the threshold value (Fig. S4).

**Fig 2:**
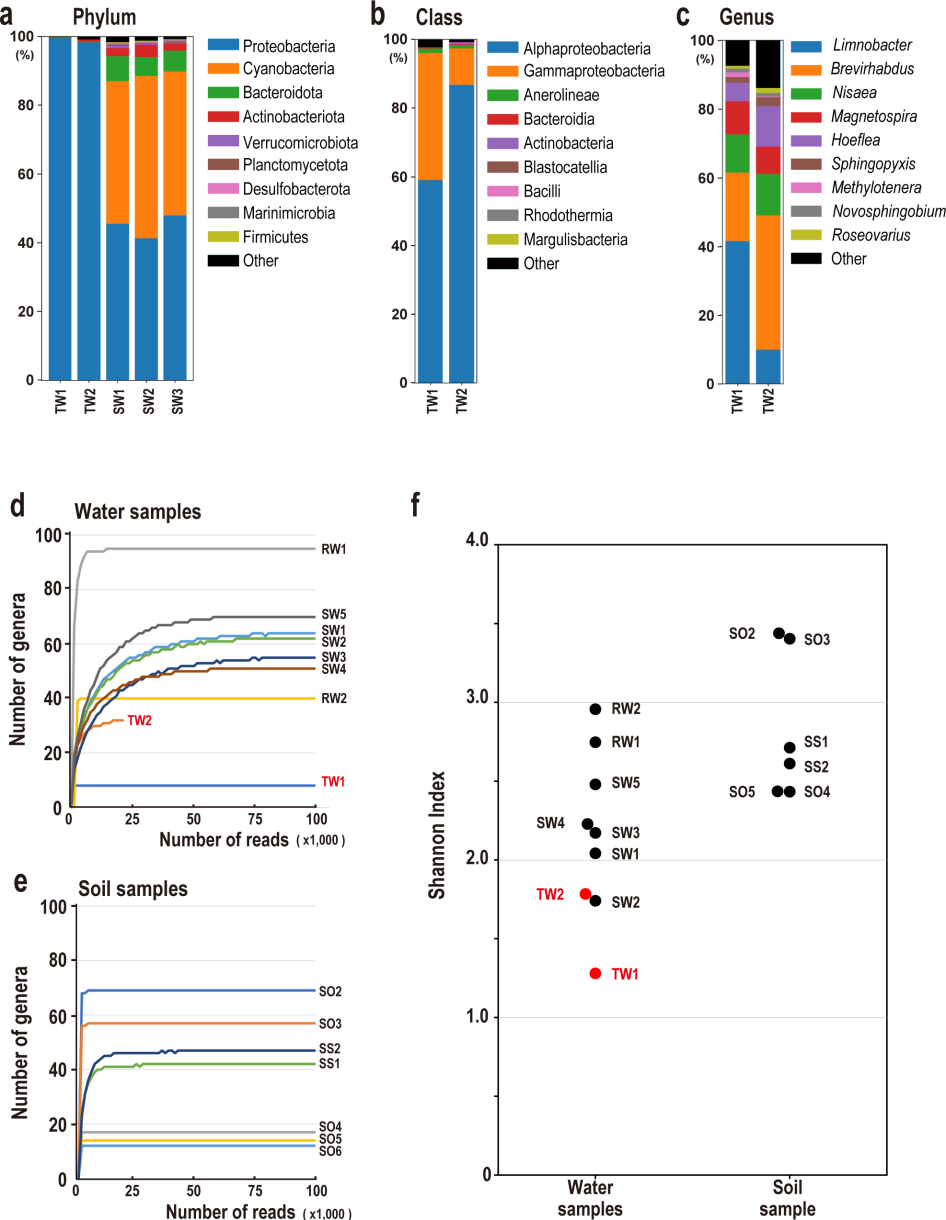
Microbial community structure analysis of torus room waters and other environmental samples. Microbial community profiles of torus room waters, TW1 and TW2, and other environmental water samples. Each figure shows the microbiota at the (**a**) phylum, (**b**) class, and (**c**) genus levels. Nucleotide sequences of the full-length 16S rRNAs in each environmental sample were determined using the Oxford Nanopore Technologies MinION sequencer. α-Rarefaction curves for each environmental sample: (**d**) water samples, including torus room waters, and (**e**) soil samples. (**f**) Comparison of microbial diversity calculated using Shannon–Wiener diversity index. Red circles indicate torus room water samples. See Table S1 for information on each sample (e.g., TW1, SW1, and RW1).

Figure 2a shows the phylum-level microbial community structure of the torus room water samples and the Fukushima seawater sample. More than 96% of bacteria in both TW1 and TW2 were members of the phylum Proteobacteria, and only a very limited group of microorganisms inhabited these environments. In the control seawater (samples SW1–SW3) collected off Fukushima Prefecture (Table S1; Fig. S2), certain proportions of species belonging to the phylum Cyanobacteria and Bacteroidota, as well as Proteobacteria, were identified. These bacteria were rarely detected in the torus room water samples. The absence of sunlight or other light in the torus room may be the reason why no Cyanobacteria were detected, although nonphotosynthetic Cyanobacteria have been reported (27). Figure 2b and c show the microbial community structures at the class and genus levels for TW1 and TW2. Table S5 presents the known microbial species (strains) that are most similar to the 16S rRNA sequences obtained for the five major microbial genera in the torus room water, together with the read counts at each of the four taxonomic levels (Fig. S5). Examination of the Proteobacteria in TW1 and TW2 showed that at the class level, Alphaproteobacteria and Gammaproteobacteria were most abundant, and most of the bacteria classified as Gammaproteobacteria were *Limnobacter thiooxidans*. The classification of microorganisms in this paper is based on the Silva 138 database (17), and some microorganisms may be classified slightly differently from those at the National Center for Biotechnology Information (NCBI). For example, Silva 138 classifies *L. thiooxidans* in Gammaproteobacteria, whereas NCBI classifies it in Betaproteobacteria. *L. thiooxidans* has been identified as a thiosulfate-oxidizing bacterium and has been reported in nuclear fuel storage (28). At the genus level, this bacterium was the predominant species in TW1, accounting for 41.6% of the total genera, whereas in TW2—the deepest area—it ranked fourth, accounting for 10.0% of the total genera. *Brevirhabdus*, which accounted for 19.9% of the total genera in TW1, was the predominant genus in TW2, accounting for 39.0% of the total genera. Among the microorganisms classified in the genus *Brevirhabdus*, the 16S rRNA sequence of *B. pacifica* was most similar to the 16S rRNA sequence obtained here (98.9% similarity, 1,326 bases long), a bacterium isolated from a hydrothermal vent (29). When we compared the microbial communities of TW1 and TW2, the estimated genera and species of microorganisms were very similar. The six most abundant genera accounted for 89.3% of the bacteria in TW1 and 83.4% of the bacteria in TW2, despite the 4-month difference in acquisition dates and the different acquisition depths. This indicates that the results obtained in this study are quite reproducible.

In this analysis, the torus room water was filtered using a 0.22-µm filter, and bacterial 16S rDNA was amplified and sequenced from the DNA prepared from the trapped microorganisms. Therefore, it is possible that microorganisms smaller than 0.22 µm were not trapped by the filter and their sequences were not detected (30). Moreover, 16S amplicon sequencing does not provide clear-cut decisions regarding the viability or growth status of the analyzed bacteria. However, the primary bacteria identified were not spore forming, and it is unlikely that DNA from dead bacteria could exist in water for up to 9 years without degradation. Furthermore, the community structure of the studied bacterial community differed from those of nearby seawater and other environmental samples (Fig. 2a), suggesting that the bacteria identified did in fact survive in the torus room water. High radiation tolerance and environmental stresses have been reported for archaea and eukaryotes, such as algae and fungi (31, 32), so future analyses of archaea and eukaryotes using targeted methods, such as metabarcoding and whole-metagenome analyses, will be required to better understand the microorganisms in torus room water environments.

### Restricted bacterial communities inhabit torus room water

To determine the number of genera within the samples, we conducted an α-diversity analysis to evaluate whether we had achieved the sequencing depth required to correctly estimate the community. An α-rarefaction curve was generated by successively extracting 1,000 random reads from the sample, counting the number of genera, and then accumulating the counts. For the diversity analysis, we used a sequence identity of 95% and a length of 1,000 bp, which was the threshold discussed in the previous section and was used to accurately estimate the genus count in the mock community. Figure 2d and e show that the genus count plateaued at ~50k reads in the aquatic samples and at ~10k reads in the soil samples, implying that there would be no further increase in the number of genera identified with the addition of more sequences. Here, we calculated the number of reads required to identify 90% of the genera in each environment: freshwater samples (RW) required ~4k reads, seawater samples (SW) required ~26k reads, soil samples (SO) required ~2k reads, seafloor soil samples (SS) required ~6k reads, and the intermediate layer (TW1) required ~1k reads. Although the deepest layer in the torus room water, TW2, had a total read count of only 16.5k, lower than those of all other samples (Table S6), no new genera were identified, even with >13k reads. From these observations, we conclude that sequencing the 16S amplicons collected in this project yielded sufficient numbers of sequences to estimate at least 90% or more of the microbial communities in the various environments.

In this study, approximately 40–95 bacterial genera were identified in seawater (SW) and freshwater (RW) samples (Table S1; Fig. S1). In contrast, eight genera were identified in TW1 and 32 genera in TW2, indicating that the number of bacterial genera identified in the torus room water was very small compared with those in other environments at Fukushima. This also suggests that the bacterial populations were more restricted in the torus room water than in the aquatic environments near Fukushima. As mentioned earlier, the dominant genera in the two torus room water samples were similar, and the six predominant bacterial genera accounted for 89.3% of bacteria in TW1 and 83.4% in TW2 (Fig. 2). Therefore, the bacteria uniquely identified in TW2 represent a small percentage of the total reads. It should be noted that, whereas all samples other than TW2 were sequenced using Flow Cell R9.4, we used Flow Cell R10.3 for TW2, which has a lower error rate, and there were 125 post-QC sequences (a threshold identity of 95% and length of 1,000 bp) for TW1 but 5,095 for TW2 (Table S6; Fig. S1). This suggests that we may have identified more relatively low abundance genera in TW2 than in TW1. Therefore, to evaluate the bacterial diversity while accounting for the difference in the post-QC read counts, we randomly sampled an equal number of sequences from TW1 and TW2. The comparison showed that the bacterial diversity was significantly higher in TW2 than in TW1 (*P* < 0.01; Fig. S6). This implies that within the torus room water, even if the overall diversity was low, there was greater bacterial diversity in the deepest part of the torus room than in the middle layer. These observations were supported by an analysis of the Shannon–Wiener diversity index, which is an α-diversity metric that considers both the number of genera and their proportions (Fig. 2f). However, it has been argued that species richness estimates are unreliable when calculated as α-diversity in cases where the species are overdispersed or the communities are dominated by rare species (32). Therefore, conclusions based solely on this index should be drawn with care.

Several interpretations of these results are possible. One hypothesis attributes the low bacterial counts in the torus room water to the bactericidal effects of hydrazine (N_2_H_4_). Following the Fukushima Daiichi NPS incident, hydrazine was introduced into the reactor of unit 2 to mitigate corrosion in the fuel pool and the corrosive effect of the reactor cooling water. (https://www.tepco.co.jp/nu/fukushima-np/images/handouts_121012_01-j.pdf). The inhibitory effects of hydrazine on microbial proliferation are well documented. The torus room is strategically situated beneath the reactor, and it is postulated that cooling water from the suppression chamber entered this compartment (https://www.nra.go.jp/data/000376560.pdf). This may account for the discrepancies in microbial diversity between TW1 and TW2 because hydrazine-enriched cooling water was retained in the upper strata of the torus room, with no potential ability to permeate to the deepest sections.

### Inferring similar environments from the microbial community structures in the torus room water

**Fig 3:**
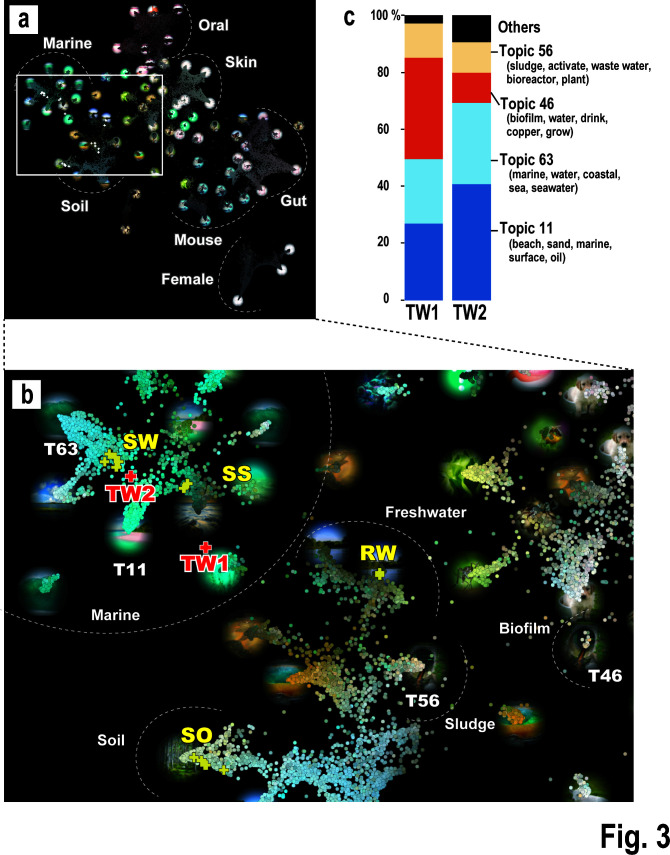
LEA of microbial communities from torus room waters and other environmental samples. (**a**) Each environmental sample was mapped onto the LEA Global Map. Overall picture of the LEA Global Map. Environments in which microorganisms were collected are indicated, such as marine, soil, oral, and skin. The circled images indicate each environmental topic, and the small dots indicate the locations of the environmental samples in the public database. The area in which the environmental samples were mapped in this study is indicated by the white box. (**b**) Expanded image of the white box region in panel a. Symbol “+” indicates the positions of the samples analyzed in this study, together with the sample names (TW1, TW2, SW, SS, RW, and SO). See Table S7 for details of each environmental topic (T11, T46, T56, and T63). (**c**) Proportion of each environmental topic estimated from the microbiota in torus room water samples TW1 and TW2.

To predict existing environments that might be similar to torus room water, we used the LEA method developed by Kurokawa et al. (22) in our analysis group (Fig. 3). LEA is an analytical approach that predicts environments from microbial communities by referencing 80 environmental “topics” (Table S7) that influence microbial community formation. The environmental topics comprise 80 representative types of environments that have been classified using topic models, with a type of statistical latent semantic analysis used in the field of natural language processing, based on metagenomic data registered on public databases (22). Figure 3a shows an overview, in which the samples from our study are plotted in two dimensions alongside samples registered in a database (LEA Global Map). The environmental samples from our study, including the torus room water, occur within the white-outlined box, which consists primarily of marine and soil samples. This region is enlarged in Fig. 3b. TW2 occurs closer to marine environments and is more proximate to the Fukushima marine samples (indicated by “SW”) than to RW, SS, or SO. In contrast, TW1 occurs closer to freshwater, biofilm, and sludge environments. When the proportions of environmental topics were examined (Fig. 3c), the main topics constituting the TW environments were marine environmental topics [topics 11 (in blue) and 63 (light blue)], together with biofilm and sludge topics in artificial environments [topics 46 (vermilion) and 56 (bright yellow)]. The combined marine environmental topics (topics 11 and 63) accounted for 69.3% of total topics in TW2, but only 48.0% of total topics in TW1, indicating a lower proportion of marine topics in TW1 than in TW2. Previous physicochemical analyses (Table S2) of the torus room water showed that TW2 was more similar to seawater compared with TW1 based on their pH and chloride ion concentrations, which is supported by the LEA analysis. The biofilm-related topic, topic 46 (vermillion), accounted for the highest proportion of bacteria in TW1 (37.4%), followed by the sludge and wastewater topic, topic 56 (bright yellow), which accounted for 9.1% of the TW1 bacteria. Furthermore, the proportion of topic 46 was higher in TW1 than in TW2 (Fig. 3c). The proportions of bacteria in topics 46 and 56 in the seawater samples (SW1–5) collected in our study were ≤0.7% (Table S7), indicating that these topics are virtually absent in typical marine samples. In conclusion, the microbial community of the torus room water contained a mixture of marine and biofilm-forming microbes, with the formation of biofilms being particularly likely in TW1.

Because biofilm formation can promote metal corrosion (as discussed below) and clog intake pipes, controlling the growth of microorganisms in torus room water is crucial. However, it is unclear whether biofilms were actually formed in the torus room water in this study because we did not directly measure the amount of biofilm formed or analyze the expression of genes associated with biofilm formation. However, as mentioned above, the torus room water at the Fukushima Daiichi NPS is highly likely to contain seawater, and seawater is known to be rich in thiosulfate (33). *Limnobacter thiooxidans* and some species of the genus *Sulfitobacter* belonging to the family Rhodobacteraceae, which were the major bacteria identified in the torus room water (see Fig. S5), are known to oxidize thiosulfates (28, 34). A recent study reported that biofilm formation in the ocean is promoted by anaerobic respiration through the oxidation of thiosulfate as an energy source (35), so we speculate that thiosulfate in the torus room water was used as a source of energy to form biofilms.

### Bacteria associated with metal corrosion in the torus room water

Some microorganisms are known to form biofilms on metal surfaces, promoting metal corrosion, a phenomenon referred to as “microbiologically influenced corrosion” (MIC) (12, 36). We investigated the bacteria associated with metal corrosion in the torus room water to understand the potential risks they pose, as suggested by our analysis of biofilm formation (Fig. 3). Microorganisms related to MIC have been categorized into six types in a previous study, based on their mechanisms of metal corrosion (12, 36). Three of these types were identified in the torus room water (Table 1): sulfur-compound oxidizers, iron/manganese oxidizers, and nitrate-reducing bacteria, all of which are aerobic bacteria. Here, we discuss the mechanism(s) of metal corrosion of each bacterial genus detected.

**TABLE 1:** Abundance of bacteria associated with metal corrosion in each environmental sample^*a*^

Types	Genus	TW1	TW2	SW1	SW2	SW3	SW4	SW5	RW1	RW2	SS1	SS2	SO2	SO3	SO4	SO5	SO6
Sulfur compound oxidizers	*Limnobacter*	41.6	10.0	−	−	−	−	−	−	0.1	0.4	−	−	−	−	−	−
	*Roseovarius*	0.8	1.5	−	−	−	−	−	−	−	−	−	−	−	−	−	−
Iron/manganese oxidizer	*Brevirhabdus*	19.9	39.0	−	−	−	−	−	−	−	−	−	−	−	−	−	−
	*Hoeflea*	5.4	11.7	−	−	−	−	−	−	−	−	−	−	−	−	−	−
	*Sphingopyxis*	1.6	2.6	−	−	−	−	−	−	−	−	−	−	−	−	−	−
	*Gallionella*	−	−	−	−	−	−	−	0.2	0.1	−	−	−	−	−	−	−
	*Leptothrix*	−	−	−	−	−	−	−	0.1	0.6	0.1	0.1	−	−	−	−	−
Nitrate reducing	*Novosphingobium*	1.1	0.9	0.1	−	−	−	−	1.2	0.8	0.2	0.6	−	−	−	−	−
	*Pseudomonas*	−	−	−	−	−	−	−	0.3	0.3	0.2	0.4	−	−	−	−	−

^*a*^ “Types” indicates the three main types of metal corrosion in which the microbial genera identified in the torus room water are involved. The distribution of the listed microbial genera in each environment is shown as the percentage (%) per environment. “−” indicates that the percentage in the environment was <0.1%. See Table S1 for details of each environment sample.

The bacterial genera identified in the torus room water and classified as sulfur compound oxidizers included *Limnobacter* (TW1, 41.6%; TW2, 10.0%; the percentages in parentheses represent their proportion in the torus room water bacteria) and *Roseovarius* (TW1, 0.8%; TW2, 1.5%). These bacteria oxidize thiosulfates and sulfur compounds (35, 37), producing H_2_SO_4_ and thereby dissolving metals such as iron and stainless steel. *L. thiooxidans* constituted the largest proportion of *Limnobacter* in TW1 (Table S5), and this species is considered to promote the oxidation of carbon steel in both marine and freshwater environments (38, 39). It is plausible that this bacterium uses the oxidation of iron found in the metals and sludge in the torus room water as an energy source. The bacterial genera identified in the torus room water that were categorized as iron- or manganese-oxidizing bacteria were *Brevirhabdus* (TW1, 19.9%; TW2, 39.0%), *Hoeflea* (TW1, 5.4%; TW2, 11.7%), and *Sphingopyxis* (TW1, 1.6%; TW2, 2.6%)(36). Among these, the genus *Brevirhabdus* was predominant in TW2 (Fig. 2c), and the predominant species of this genus, *B. pacific* (see Table S5), has been reported to oxidize manganese (40). When the manganese oxides formed adhere to the surfaces of stainless steel pipes, they create an electric potential difference, thus exacerbating corrosion (41). The genera *Hoeflea* and *Sphingopyxis* are classified as iron-oxidizing bacteria, oxidizing divalent iron ions (Fe^2+^) to trivalent iron ions (Fe^3+^) and producing reddish-brown ferric hydroxide [Fe(OH)_2_] (42). The nitrate-reducing bacterial genus *Novosphingobium* was also detected in the torus room water (TW1, 1.1%; TW2, 0.9%). Although the proportion of this genus in the TW samples was low and it was not among the predominant bacteria, corrosion by nitrate-reducing bacteria has a substantial impact. During nitrate reduction, the NO_2_^−^ produced induces corrosion (43) and can extract electrons from the surface of carbon steel (36, 43).

In summary, the proportions of total genera of these MIC-associated bacterial genera in the torus room water were 70.4% for TW1 and 65.7% for TW2. In contrast, their proportions in the water samples analyzed in this study (other than those from the torus room water) were ≤1.2%, suggesting that the majority of bacteria present in the torus room water were potentially associated with MIC. Further examination of the impact of each of these genera on metal corrosion in the future is essential.

### Identification of radiation-resistant bacteria in torus room water

The ^137^Cs concentrations in the samples of seawater and seabed soil collected from the vicinity of Fukushima were ≤500 Bq/kg, whereas those in the torus room water were 1.3 × 10^9^ Bq/L for TW1 and 1.4 × 10^9^ Bq/L for TW2, indicating that the torus room water contained substantially higher levels of the radioactive substance ^137^Cs (Table S1). Therefore, we analyzed the proportion of known radiation-resistant bacteria in the various samples. The radiation-resistant bacteria detected in the torus room water consisted of the genera *Methylobacterium* (0.2% of the total bacteria), *Brevundimonas* (< 0.1%), and *Massilia* (< 0.1%) (Table S8), but these bacteria were not predominant in the microbial communities in the torus room water. In fact, the proportions of radiation-resistant bacteria in the torus room water were the same as those in the environmental samples collected from Fukushima Prefecture.

Our understanding of the radiation resistance of the bacteria identified in torus room water is currently extremely limited. Therefore, we tested the radiation resistance of the most abundant bacterial species detected in TW1 and its closest relative (see Table S5), *L. thiooxidans* strain CS-K2 (with a 16S rRNA sequence similarity of ≥98.1%). When subjected to γ-irradiation at doses of 0, 2.5, and 5.0 Gy/h for 6 days, the number of colonies of these species decreased significantly compared with those of the corresponding nonirradiated samples: approximately one-tenth for the sample irradiated with 0.36 kGy (2.5 Gy/h) and approximately one-hundredth for the sample irradiated with 0.72 kGy (5.0 Gy/h) (Fig. S7). The previously reported *D*_10_ values, which represent the radiation dose at which the survival rate drops to 10%, are *Escherichia coli* (normal bacteria) 0.15 kGy, *Staphylococcus aureus* (normal bacteria) 0.21 kGy, *Methylobacterium radiotolerans* (radiation-resistant bacterium) 1.4 kGy, and *Deinococcus radiodurans* (radiation-resistant bacterium) 10 kGy (44). The *D*_10_ value of *L. thiooxidans* strain CS-K2, as determined in this experiment, was 0.36 kGy, demonstrating that its radiation resistance was similar to those of normal bacteria and that it was not exceptionally resistant to radiation. In contrast, it has been reported that *E. coli* displays high radiation resistance, attributed to genomic changes, after repeated radiation exposure (45). Therefore, it cannot be excluded that that bacterial species similar to *L. thiooxidans*, which had survived in the torus room for approximately 9 years, had acquired some degree of radiation resistance. Further research is required to identify mutations in the genomes of the torus room-derived microorganisms and their radiation resistance.

This discussion also holds true for the environmental samples from the surface soils and seawater near the Fukushima Daiichi NPS, which were exposed to significantly lower radiation levels than the torus room water. Specifically, when we compared the microbial community structures in the surface soils (SO4–SO6) within approximately 1 km of Fukushima Daiichi NPS (which displayed a radiation level of 48.2 µSv/h) with those of soils in Yamakiya, located ~40 km from Fukushima Daiichi NPS, which displayed a radiation level of 0.1 µSv/h (samples MC1A and MC1B), both community profiles predominantly contained the phyla Proteobacteria and Acidobacteria and showed remarkable similarities (Fig. S8). Furthermore, a hierarchical clustering analysis and principal coordinates analysis indicated that the soils collected in this study (SO4–SO6) and the soils from Yamakiya (MC samples) formed closely related clusters (Fig. S9), suggesting that there were no substantial differences in the microbial community structures that could be attributable to differences in radiation. Moreover, in a microbial community analysis of soils with varying radiation contamination levels within Fukushima Prefecture by Ihara et al., no significant differences in the microbial communities were observed, and radiation-resistant bacteria did not constitute a major bacterial group in these soil samples (11).

### Commonality and co-detection ratios of bacteria identified in the torus room water

**Fig 4:**
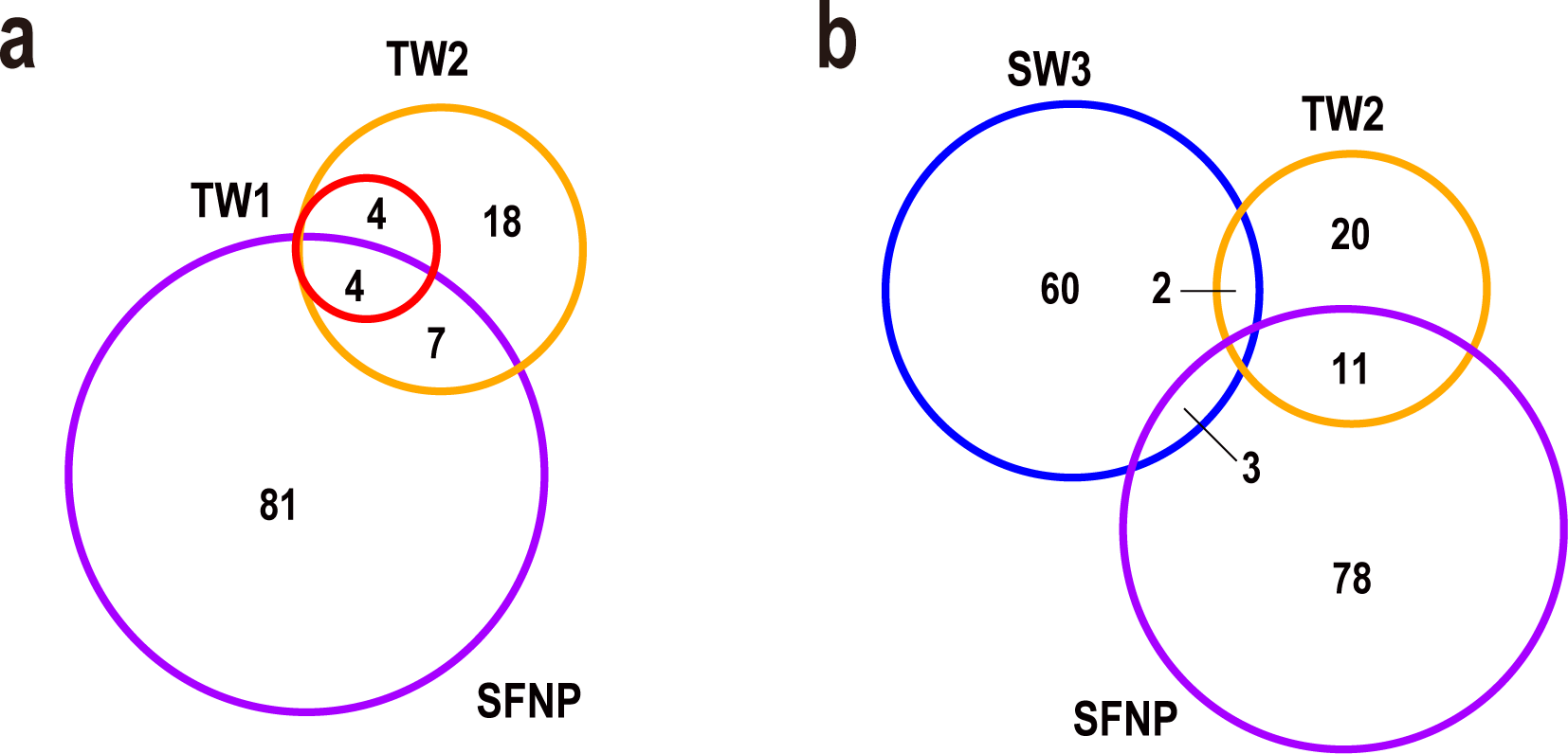
Number of commonly identified bacterial genera in each environmental sample. Venn diagram showing the numbers of bacterial genera commonly found in the torus room waters (TW1 and TW2), seawater offshore from Fukushima Daiichi NPS (SW3), and a spent nuclear fuel pool (SNFP). (**a**) numbers of common bacterial genera in TW1, TW2, and an SNFP. (**b**) Numbers of common bacterial genera in TW2, an SNFP, and SW3.

Biofilms host multiple species of bacteria, which are known to be resistant to various environmental stresses (46, 47). To analyze the symbiotic relationships among these bacteria, the commonality of the bacterial genera in the torus room water and environmental samples was examined. We included a data set of information from studies of bacterial genera identified in spent nuclear fuel pools (SNFPs) (48–54), an environment considered similar to torus room water. As shown in Fig. 4, the bacterial genera identified in the torus room water (TW1 and TW2) shared more similarities with those identified in the SNFPs than with those in Fukushima seawater (SW3). In Fig. 4a, the eight genera identified in TW1 were contained within the 33 genera identified in TW2. Moreover, four bacterial genera were identified in SNFPs, TW1, and TW2 (*Limnobacter*, *Sphingopyxis*, *Methylotenera*, and *Novosphingobium*). Excluding these four, only seven bacterial genera were common to SNFP and TW2 (*Massilia*, *Methylobacterium*, *Cutibacterium*, *Brevundimonas*, *Sphingobium*, *Pseudonocardia*, and *Porphyrobacter*). Of these seven genera, three (*Brevundimonas*, *Methylobacterium*, and *Massilia*) reportedly contain radiation-resistant bacteria (55). However, these three genera constituted <0.2% of the total bacteria and were not the predominant bacteria in the environment (Table S8). In Fig. 4a and b, 50.0% of bacterial genera identified in TW1 were also identified in SNFP (4 of 8 genera), as were 33.3% (11 of 33 genera) of the genera identified in TW2, whereas only 4.6% of genera (2 of 65 genera) in SW3 were detected in SNFP. This suggests that the environments of nuclear-related facilities, specifically in France, Brazil, the United Kingdom, and Spain (48–54), share common bacterial genera (Fig. S10).

**Fig 5:**
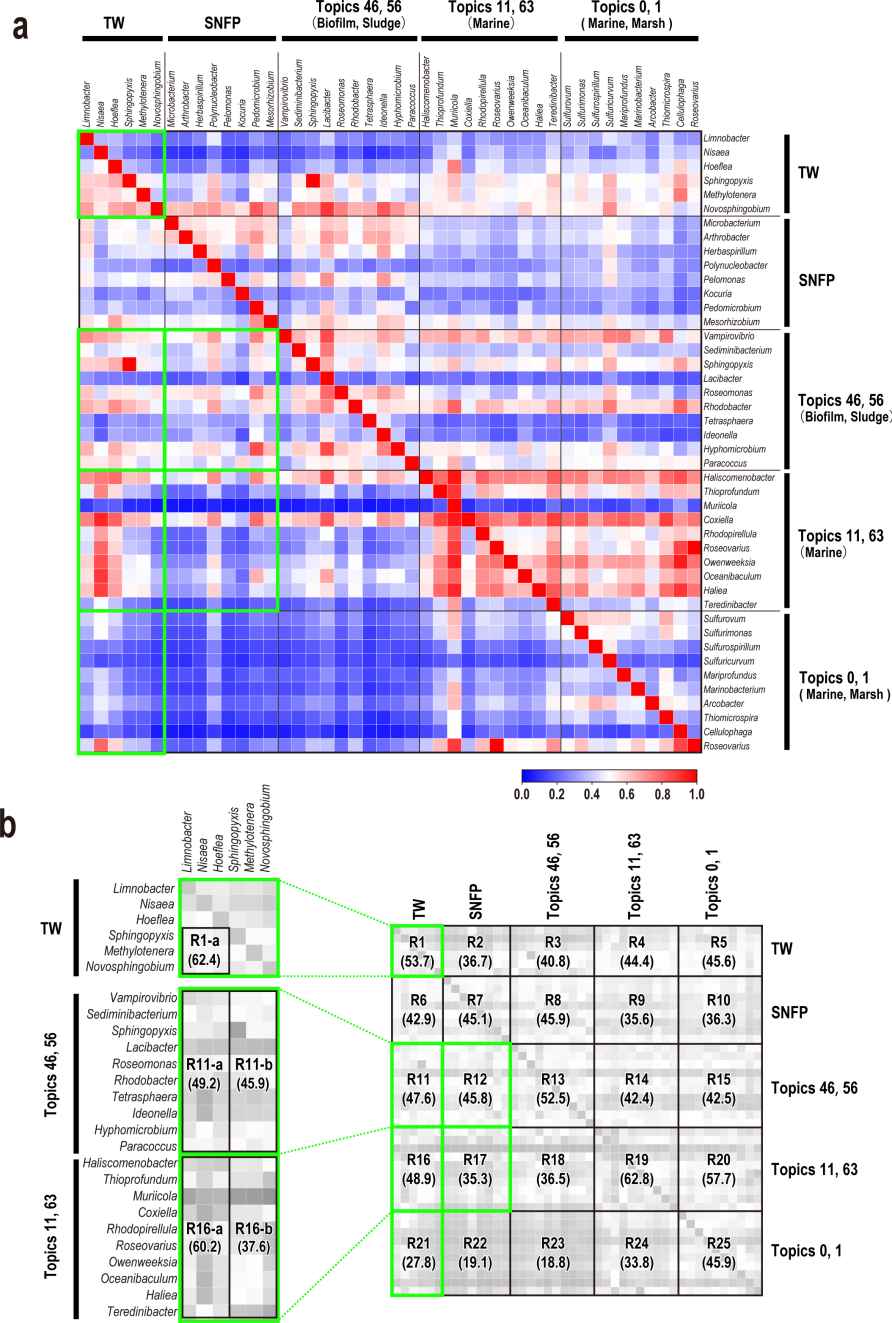
Heatmap of co-detection rates for microbial genera in MicrobeDB. (**a**) Heatmap of co-detection rates of microbial genera. The heatmap in blue–red shows the rates of co-detection of the microbial genera shown on the horizontal and vertical axes for each microbe registered in MicrobeDB.jp (see Materials and Methods). Increasing rates of co-detection are indicated from blue to red on the heatmap. The bacterial genera were classified roughly into the following five categories: (i) major bacterial genera identified in torus room waters (TW), (ii) eight genera randomly selected from 81 genera identified specifically in SNFPs (SNFP), (iii) major bacterial genera belonging to the most abundant topics (topics 46 and 56) represented in the torus room waters (see Fig. 3c), (iv) major bacterial genera belonging to the most abundant topics (topics 11 and 63) represented in the torus room waters (see Fig. 3c), and (v) major bacterial genera belonging to topics least relevant to the torus room water (topics 0 and 1). See Table S7 for details of each environmental topic. (**b**) Grayscale heatmap (right) shows the area names and the average co-detection rates (percentages) according to the environment of the microbial genera shown in panel **a**. The heatmap (left) zooms in on R1, R11, and R16 and shows the average co-detection rate (percentages) in each part within the region.

So far, we have compared the community structures within restricted environments at the genus level. To gain insight into the habitats of the bacterial genera detected in the torus room water, we used the MicrobeDB.jp database (22), which holds about 60,000 metagenomic data, to analyze the situations in which two specific genera were detected together. In this analysis, we calculated the proportion of the bacterial genera indicated in the columns that were included in the metagenomic data, where the bacteria genera shown in rows are contained (Fig. 5). The results are presented as a heatmap in Fig. 5a. Here, the bacterial genera are divided into the following five categories: (i) the six most abundant bacterial genera identified in TW; (ii) eight bacterial genera identified in SNFPs; (iii) eight bacterial genera constituting the biofilm/sludge environment topic, a major topic in the torus room water environment (topics 46 and 56); (iv) eight bacterial genera constituting the marine environment topic, another major topic in the torus room water environment (topics 11 and 63); and (v) eight bacterial genera constituting topics that were barely detected in the torus room water environment (topics 0 and 1). In Fig. 5a, the torus room water does not include the genera *Brevirhabdus* or *Magnetospira*, which were relatively abundant in the torus chamber but are not registered in MicrobeDB.jp. The four main topics of bacteria detected in the torus room water are divided into topics 46–56 and 11–63 based on the distances on the LEA Global Map (see Fig. 3). To enhance the clarity of Fig. 5a, we partitioned the heatmap into distinct regions for convenience and assigned labels from R1 through R25 to each of these regions (Fig. 5b).

To confirm the validity of the analysis, we focused on the rows that include the main torus room water topics (topics 11, 46, 56, and 63) and the topics barely detected in the torus room water (topics 0 and 1), as identified in the previous analysis (Fig. 3) (R11, R16, and R21 in Fig. 5b). For example, R11 shows the co-detection rates for the major genera constituting the biofilm/sludge environment topic (topics 46 and 56) for the most abundant bacterial genera identified in the torus room water. The average co-detection rates for R11 and R16 were 47.6% and 48.9%, respectively, whereas the average co-detection rate for R21 was 27.8%, confirming that this co-detection rate was lower than those for R11 and R16. These results indicate that the co-detection rate was high for the bacterial genera constituting the four main topics detected in the torus room water (topics 11, 46, 56, and 63), whereas the co-detection rate was low for the microbes constituting topics that were barely detected in the torus room water (topics 0 and 1). These results would support the results of Fig. 3c.

When we further examined parts of areas R11, R16, and R21, R1-a (shown on the left in Fig. 5b) was surrounded by three columns (genera *Limnobacter*, *Nisaea*, and *Hoeflea*) and three rows (genera *Sphingopyxis*, *Methylotenera*, and *Novosphingobium*) with high co-detection rates within R1. R1-a had an average co-detection rate (62.4%) higher than the overall average of R1 (53.7%; Fig. 5b), suggesting that these microbes are more likely to inhabit the same environment. A previous study (56) identified the three genera *Limnobacter*, *Sphingopyxis*, and *Novosphingobium* in the same biofilms and crude oil, supporting the validity of this analysis. Similarly, in areas R11 and R16, it is observed that it is divided into groups with relatively high (R11-a and R16a) and low (R11-b and R16b) co-detection rates. Here, R11-a and R16-a contained the genera *Limnobacter*, *Nisaea*, and *Hoeflea* in TW, while R11-b and R16-b contained *Sphingopyxis*, *Methylotenera*, and *Novosphingobium* in TW. The average co-detection rates for the biofilm and sludge environments (topics 46 and 56) against the torus room water (R11-a and R11-b) were 49.2% and 45.9%, respectively, indicating similar co-detection rates. In contrast, the average co-detection rates for marine topics (topic 11 and 63) against the torus room water (R16-a and R16-b) were 60.2% and 37.6%, respectively, indicating a lower co-detection rate in R16-b than in R16-a. From these results, it is clear that the three most abundant genera identified in the torus room water (*Limnobacter*, *Nisaea*, and *Hoeflea*) are bacteria that occur frequently in both biofilm and sludge environments and in marine environments, whereas the other three genera (*Sphingopyxis*, *Sphingopyxis*, and *Novosphingobium*), which are detected at the same levels as the three most abundant genera in biofilm and sludge environments, are less frequently detected in marine environments. This trend was the same for the SNFP column, where R17 was lower than R12, suggesting that the bacterial genera identified in SNFP occur within biofilms but are not abundant in seawater.

To sum up, nuclear-related facilities, such as torus room water and SNFPs, contain bacterial communities commonly detected in biofilms and sludge. However, the three genera constituting a large proportion of the microbial community in the torus room water (*Limnobacter*, *Nisaea*, and *Hoeflea*) also occur abundantly in marine environments. From this perspective, it can be said that the bacterial community in the torus room water was unique, relative to those in normal seawater or in nuclear-related facilities. Although speculative, possible sources of these bacterial groups were (i) bacteria that flowed in with the tsunami or injected seawater and survived in the torus chamber environment or (ii) bacteria adapted to the marine environment that had flowed into the torus room from the damaged part of the suppression chamber in the treated water circulating in the reactor.

### Conclusions

In this study, radiation-contaminated waters, lying stagnant in a semi-enclosed environment in the torus room of Fukushima Daiichi NPS for approximately 9 years, were collected from two different depths (TW1 and TW2), and a comprehensive analysis of the bacterial communities was conducted based on 16S rRNA sequences. These bacterial communities showed lower diversity than those in environmental samples collected in Fukushima Prefecture. The torus room water bacterial communities are presumed to have originated from a mixed environment of natural marine microbial groups and the bacterial groups in biofilms and sludge generated in artificial environments. In both TW1 and TW2, the proportions of bacterial genera known to be radiation resistant were extremely low, suggesting that the impact of radioactivity on selection within the torus room water was minimal. In contrast, ~70% of the bacterial genera in the torus room water were associated with metal corrosion, indicating that the impact of bacteria on metal corrosion must be considered in long-term decommissioning work. Some of the bacterial genera detected were the same as those previously identified in SNFPs, but those adapted to marine environments tended to be more abundant in the torus room water than in SNFPs.

## Data Availability

The data supporting the findings of this study are available within the paper and its supplemental material. All relevant accession numbers are reported in the supplemental tables.

## SUPPLEMENTAL MATERIAL

Supplemental materials aem.02113-23-s0001.pdf Supplemental Tables S1 to S8, supplemental Figures S1 to S10.

ASM does not own the copyrights to Supplemental Material that may be linked to, or accessed through, an article. The authors have granted ASM a non-exclusive, world-wide license to publish the Supplemental Material files. Please contact the corresponding author directly for reuse.
