# Supplementary material for: Microbiome analysis of the restricted bacteria in radioactive element-containing water at the Fukushima Daiichi Nuclear Power Station
Source: Appl Environ Microbiol. 2024 Mar 12;90(4):e02113-23. doi: 10.1128/aem.02113-23 (PMC11022576; doi:10.1128/aem.02113-23)
Supplement: Supplemental materials — Supplemental Tables S1 to S8, supplemental Figures S1 to S10. [file aem.02113-23-s0001.pdf]

Supplementary Materials

Microbiome analysis of the restricted bacteria in radioactive-element-containing water at the Fukushima Daiichi Nuclear Power Station

Tomoro Warashina,[a,b] Asako Sato,[a] Hiroshi Hinai,[c] Nurislam Shaikhutdinov,[d] Elena Shagimardanova,[d,e,f] Hiroshi Mori,[g] Satoshi Tamaki,[a] Motofumi Saito,[a,b] Yukihisa Sanada,[c] Yoshito Sasaki,[c] Kozue Shimada,[c] Yuma Dotsuta,[c] Toru Kitagaki,[c] Shigenori Maruyama,[h] Oleg Gusev,[d,e,i] Issay Narumi,[j] Ken Kurokawa,[g] Teppei Morita,[a,b] Toshikazu Ebisuzaki,[k] Akihiko Nishimura,[c] Yoshikazu Koma,[c] Akio Kanai[a,b,l]#

[a]Institute for Advanced Biosciences, Keio University, Tsuruoka, Japan

[b]Systems Biology Program, Graduate School of Media and Governance, Keio University, Fujisawa, Japan

[c]Japan Atomic Energy Agency, Tokai, Japan

[d]Regulatory Genomics Research Center, Institute of Fundamental Medicine and Biology, Kazan (Volga Region) Federal University, Kazan, Russia

[e]Life Improvement by Future Technologies (LIFT) Center, Skolkovo, Moscow, Russia

[f]Loginov Moscow Clinical Scientific Center, Moscow, Russia

[g]National Institute of Genetics, Mishima, Japan

[h]Earth-Life Science Institute, Tokyo Institute of Technology, Tokyo, Japan

[i]Intractable Disease Research Center, School of Medicine, Juntendo University, Tokyo, Japan

[j]Faculty of Life Sciences, Toyo University, Oura-gun, Japan

[k]Computational Astrophysics Laboratory, RIKEN, Wako, Japan

[l]Faculty of Environment and Information Studies, Keio University, Fujisawa, Japan

Running Head: Microbiota from Fukushima Nuclear Power Station

# Address correspondence to Akio Kanai, akio@sfc.keio.ac.jp

This PDF file includes:

Supplementary Tables S1 to S8

Supplementary Fig. S1 to S10

Supplementary Table S1. Summary of environmental sample information

ID	Environment	Coordinate	Place	Description	BioSample ID	Collection date	Depth m	mSv/h (1m from ground)	Bq ^{137}Cs /kg	pH	Ref.
TW1	Torus room water	37°25'20.1"N 141°01'57.7"E	Fukushima	Unit2 torus room	SAMD00571327	2020.02.13	0.3-1.0 m from the bottom	N/A	1.3×10^9	7.42	This study
TW2	Torus room water	37°25'20.1"N 141°01'57.7"E	Fukushima	Unit2 torus room	SAMD00571328	2020.06.30	Bottom	N/A	1.4×10^9	7.78	This study
SO1	Soil	37°24'59.3" N 141°01'24.7"E	Fukushima	Surface soil 1	SAMD00571322	2019.08.19	0.00 - 0.01	48.2	N/A	5.9	This study
SO2	Soil	37°24'59.3" N 141°01'24.7"E	Fukushima	Surface soil 2	SAMD00567467	2019.08.19	0.00 - 0.01	48.2	7.5×10^6	5.9	This study
SO3	Soil	37°24'59.3" N 141°01'24.7"E	Fukushima	Surface soil 3	SAMD00571323	2019.08.19	0.00 - 0.01	48.2	N/A	5.9	This study
SO4	Soil	37°24'59.3" N 141°01'24.7"E	Fukushima	0-2 cm deep from the ground (Fukushima)	SAMD00571324	2019.08.19	0.00 - 0.02	N/A	3.1×10^6	5.9	This study
SO5	Soil	37°24'59.3" N 141°01'24.7"E	Fukushima	6-7 cm deep from the ground (Fukushima)	SAMD00571325	2019.08.19	0.06 - 0.07	N/A	8.7×10^2	5.9	This study
SO6	Soil	37°24'59.3" N 141°01'24.7"E	Fukushima	11-13 cm deep from the ground (Fukushima)	SAMD00571326	2019.08.19	0.11 - 0.13	N/A	1.7×10^3	5.9	This study
SW1	Seawater	37°29'27.6" N 141°02'58.9"E	Fukushima	Seawater on the seabed (off the Kodaka River)	SAMD00571329	2019.12.19	0	N/A	100 - 250	8.4	This study
SW2	Seawater	37°35'4.8" N 141°03'57.5"E	Fukushima	Seawater on the seabed (off the Ukedo River)	SAMD00571330	2019.12.19	N/A	N/A	20 - 40	8.5	This study
SW3	Seawater	37°25'27.5" N 141°03'11.3"E	Fukushima	Surface water near Fukushima Daiichi Nuclear Power Station	SAMD00571331	2019.12.19	N/A	N/A	250 - 500	8.2	This study
SW4	Seawater	36°29.7'N 140°38.8'E	Ibaraki	Surface water (Hitach offshore)	SAMD00571332	2020.02.06	0	N/A	N/A	6.2	This study
SW5	Seawater	36°29'08"N 140°36'45"E	Ibaraki	Surface water (Hitach port)	SAMD00571333	2020.02.06	0	N/A	N/A	6.2	This study
SS1	Seabed soil	37°29'27.6" N 141°02'58.9"E	Fukushima	Seabed soil (off the Kodaka River)	SAMD00571334	2019.12.19	N/A	N/A	45	5.6	This study
SS2	Seabed soil	37°35'4.8" N 141°03'57.5"E	Fukushima	Seabed soil (off the Ukedo River)	SAMD00571335	2019.12.19	N/A	N/A	356	5.6	This study
RW1	River water	37°09'50.3"N 140°25'08.9"E	Fukushima	River water 1 (Yashiro river)	SAMD00571336	2020.03.17	0	N/A	N/A	6.2	This study
RW2	River water	37°09'50.3"N 140°25'08.9"E	Fukushima	River water 2 (Yashiro river)	SAMD00571337	2020.10.29	0	N/A	N/A	5.6	This study
MC1	Mock community	N/A	N/A	ZymoBIOMICS Microbial Community Standard(D6300)	N/A	N/A	N/A	N/A	N/A	N/A	This study
MC1A	Soil	37°35'40.8"N 140°40'38.2"E	Fukushima	MC1 (Yamakiya)	SRX4803261	2014.07	0.03	1.0 - 2.0	34,430	5.8	[1]
MC1B	Soil	37°35'40.8"N 140°40'38.2"E	Fukushima	1 ReplicateMC1 (Yamakiya)	SRX4803260	2014.07	0.03	1.0 - 2.0	34,430	5.8	[1]
MC1C	Soil	37°35'40.8"N 140°40'38.2"E	Fukushima	2 ReplicateMC1 (Yamakiya)	SRX4803354	2014.07	0.2	1.0 - 2.0	860	5.8	[1]
MC1D	Soil	37°35'40.8"N 140°40'38.2"E	Fukushima	3 ReplicateMC1 (Yamakiya)	SRX4803355	2014.07	0.2	1.0 - 2.0	860	5.8	[1]
MC2B	Soil	37°35'40.8"N 140°40'38.2"E	Fukushima	MC2 (Yamakiya)	SRX4803356	2014.07	0.08	1.0 - 2.0	4,320	5.8	[1]
MC2C	Soil	37°35'40.8"N 140°40'38.2"E	Fukushima	1 ReplicateMC2 (Yamakiya)	SRX4803357	2014.07	0.08	1.0 - 2.0	4,320	5.8	[1]
MC2D	Soil	37°35'40.8"N 140°40'38.2"E	Fukushima	2 ReplicateMC2 (Yamakiya)	SRX4803358	2014.07	0.08	1.0 - 2.0	4,320	5.8	[1]

The upper section presents the sample information obtained in the current study, and the lower section provides the sample details (MC1A–MC2D) reported by Yamakiya in a previous study (Ref. 1). The location of Yamakiya's samples is indicated by the coordinates of the center of the area.

Reference:

1. Hoyos-Hernandez, C. *et al.* Community structure and functional genes in radionuclide contaminated soils in Chernobyl and Fukushima. *FEMS Microbiol Lett* **366**, 180 (2019).

Supplementary Table S2. Physicochemical information on torus room waters

a Information on torus room waters TW1 and TW2 used in this study and on their radioactive elements

	TW1	TW2
**Collection date**	2020.02.13	2020.06.30
**Place**	Unit 2 Torus Room	Unit 2 Torus Room
	0.3 m and 1.0 m from the bottom	Bottom water
**Volume**	30 mL	15 mL
**pH**	7.42	7.78
**Oxidation-Reduction Potential**	420.2 mV	396.9 mV
**Electrical conductivity**	4.68 S/m	N/A
**chloride ion concentration**	14,000 ppm	20,000 ppm
**Radioactivity in contaminated water***		
**Pu-238**	4.6×10^{0} (Bq/L)	5.2×10^{3} (Bq/L)
**Pu-239 + 240**	1.6×10^{0} (Bq/L)	1.8×10^{3} (Bq/L)
**Cs-134**	7.5×10^{7} (Bq/L)	9.3×10^{7} (Bq/L)
**Cs-137**	1.3×10^{9} (Bq/L)	1.4×10^{9} (Bq/L)
**Co-60**	4.6×10^{3} (Bq/L)	1.8×10^{6} (Bq/L)

*Date for decay correction: 2020 Feb 13, 2020 Jun 30

b Amount of each element contained in torus room waters TW1 and TW2

	TW1		TW2	
	**Residue (mg/mL)**	**Filtrate (mg/mL)**	**Residue (mg/mL)**	**Filtrate (mg/mL)**
**B**	$< 1.0\times10^{-3}$	$< 2.0\times10^{1}$	4.6×10^{-3} (0.18%)	$< 9.0\times10^{0}$
**Na**	1.3×10^{-1} (88.2%)	7.5×10^{3} (83.2 %)	$< 1.0\times10^{-2}$	8.3×10^{3} (79.5%)
**Mg**	1.1×10^{-2} (7.3%)	8.5×10^{2} (9.4 %)	4.2×10^{-2} (1.6%)	1.3×10^{3} (12.5%)
**Al**	$< 1.0\times10^{-3}$	N/A	1.1×10^{-2} (7.1%)	N/A
**Si**	$< 1.0\times10^{-3}$	$< 2.0\times10^{1}$	2.7×10^{-2} (1.1%)	$< 9.0\times10^{0}$
**K**	$< 1.0\times10^{-3}$	2.7×10^{2} (3.0 %)	$< 1.0\times10^{-2}$	3.3×10^{2} (3.2%)
**Ca**	5.4×10^{-3} (3.6%)	3.9×10^{2} (4.3 %)	2.0×10^{-2} (0.79%)	5.1×10^{2} (4.9%)
**Ti**	$< 1.0\times10^{-3}$	N/A	$< 1.0\times10^{-2}$	N/A
**Mn**	$< 1.0\times10^{-3}$	$< 2.0\times10^{1}$	6.4×10^{-3} (0.25%)	$< 9.0\times10^{0}$
**Fe**	1.3×10^{-3} (0.8%)	$< 2.0\times10^{1}$	2.0×10^{0} (78.2%)	$< 9.0\times10^{0}$
**Zn**	$< 1.0\times10^{-3}$	N/A	2.8×10^{-1} (10.7%)	N/A
**Sr**	$< 1.0\times10^{-4}$	4.5×10^{0} (0.05%)	$< 1.0\times10^{-3}$	5.7×10^{0} (0.05%)

N/A: not applicable

Supplementary Table S3. List of PCR primers used in this study

For Nanopore sequencing

Name	Sequence	Comment
27F	5'-AGAGTTTGATCMTGGCTCAG-3'	Bacterial 16S rRNA primers for V1-V9 region (Ref. 1)
1492R	5'-TACGGYTACCTTGTTAYGACTT-3'	
lu_16S_8F_v7	5'-CAAGCAGAAGACGGCATACGAGAT NNNYRNNNYRNNNYRNNN AGRGTTYGATYMTGGCTCAG-3'	Bacterial 16S rRNA primers for V1-V9 region (Ref. 1)
lu_16S_1391R_v7	5'-AATGATACGGCGACCACCGAGATC NNNYRNNNYRNNNYRNNN GACGGGCGGTGWGTRCA-3'	
lu_pcr_fw_v7	5'-CAAGCAGAAGACGGCATACGAGAT-3'	Primer for library amplification (Ref. 2)
lu_pcr_rv_v7	5'-AATGATACGGCGACCACCGAGATC-3'	

For illumina sequencing

Name	Sequence	Comment
341f	5'-CCTACGGGNGGCWGCAG-3'	Bacterial 16S rRNA primers for V3-V4 region (Ref. 3)
785r	5'-GACTACHVGGGTATCTAATCC-3'	

References:

1. Oxford Nanopore Technologies. 16S Barcoding Kit. https://store.nanoporetech.com/16s-barcoding-kit-1-24.html (2023).
2. Karst, S. M. *et al.* High-accuracy long-read amplicon sequences using unique molecular identifiers with Nanopore or PacBio sequencing. *Nature Methods* **18**, 165–169 (2021).
3. Klindworth, A. *et al.* Evaluation of general 16S ribosomal RNA gene PCR primers for classical and next-generation sequencing-based diversity studies. *Nucleic Acids Res* **41**, (2013).

Supplementary Table S4. Information on environmental DNA preparation from torus room water samples and DNA sequencing

	TW1		TW2	
**DNA extraction summary**				
Amount of torus room water	30.	mL	15.	mL
Amount of extracted DNA	9.	ng	4.	ng
Amount of DNA used for PCR	1.34	ng	0.08	ng
Amount of DNA used for sequencing	8.7	ng	21.4	ng
**Sequencing equipment information**				
Flow cell type	FLO-MIN106 (R9.4)		FLO-MIN111 (R10.3)	
library preparation kit	SQK-RAB204		SQK-LSK109	
Guppy version	3.2.9		5.0.17	
**Sequencing summary**				
Mean read length	1,412.4		1,419.4	
Mean read quality	10.8		12.3	
Median read length	1,434		1,415	
Median read quality	10.7		12.2	
Number of reads	1,026,901		16,518	
Read length N50	1,434		1,416	
Total bases	1,471,933,643		23,445,386	

Supplementary Table S5. Examples of known species classified into the five genera with the highest relative abundance in torus room waters and their numbers of sequence reads

Organism			Number of Sequences							
			TW1				TW2			
Genus	Species (Strain)	NCBI GenBank accession no.	>97%	>95%	>90%	>85%	>97%	>95%	>90%	>85%
Limnobacter	*Limnobacter_thiooxidans*	AJ289885	0	61	23509	27476	25	344	730	746
	uncultured_organism	HQ768593	0	0	274	358	0	0	2	2
	Limnobacter_thiooxidans	GQ284439	0	0	195	321	22	245	610	617
	uncultured_bacterium	DQ860042	0	0	117	175	0	0	6	9
	uncultured_Limnobacter	KF851215	0	0	111	173	0	1	5	5
Brevirhabdus	*Brevirhabdus_pacifica*	CP019124	0	36	8436	9769	264	2435	5933	6026
	Brevirhabdus_pacifica	KC018457	0	5	2365	2579	0	0	0	0
	Brevirhabdus_pacifica	KF356416	0	2	1485	1625	0	0	0	0
	uncultured_bacterium	AB750585	0	0	194	244	0	0	13	15
Nisaea	uncultured_OM75	JX526777	0	0	3194	4366	0	1	8	8
	bacterium_L12	AY345389	0	0	1920	2637	0	14	65	70
	Nisaea_nitritireducens	DQ665839	0	0	1516	2017	9	423	1699	1760
	*Nisaea_* sp.	AM990885	0	0	177	232	0	0	1	3
	uncultured_bacterium	JQ712075	0	0	41	130	0	0	3	3
Magnetospira	rock_porewater	LADL02000502	0	7	5514	6591	48	426	1204	1249
	alpha_proteobacterium	JQ085423	0	0	327	674	0	1	9	14
	uncultured_marine	FJ826317	0	0	278	483	0	0	0	0
	uncultured_nuHF1	GU568015	0	0	0	32	0	0	0	1
	uncultured_bacterium	KJ590638	0	0	5	15	0	0	1	2
Hoeflea	*Hoeflea_alexandrii*	AJ786600	0	4	1871	2316	27	419	1456	1486
	Hoeflea_olei	LQZT01000024	0	1	467	572	5	55	239	247
	Antarctic_bacterium	AJ441009	0	0	299	393	0	0	2	2
	uncultured_Hoeflea	JX529413	0	0	149	219	0	2	13	15
	Hoeflea_olei	HG965759	0	0	115	143	0	0	0	0

Supplementary Table S6. Summary of the number of reads obtained using next-generation sequencers from each environment

a Summary of 16S amplicon sequencing using an Oxford Nanopore Technologies MinION sequencer

ID	Accession No. (BioSample ID)	# of QC-passed reads (Guppy)	# of QC-passed reads (Porechop)	# of sequences after random sampling	# of classified reads		
					default	identity > 90	identity > 95
TW1	SAMD00571327	2,052,063	1,026,901	99,874	99,874	62,889	139
TW2	SAMD00571328	49,735	16,518	16,518	16,494	15,292	5,093
SO1	N/A	N/A	N/A	N/A	N/A	N/A	N/A
SO2	SAMD00567467	1,514,351	345,610	99,697	99,696	49,906	334
SO3	SAMD00571323	540,712	443,340	99,794	99,794	50,026	379
SO4	SAMD00571324	598,421	313,788	99,822	99,821	41,806	90
SO5	SAMD00571325	931,896	494,766	99,894	99,892	40,714	124
SO6	SAMD00571326	940,312	498,271	99,908	99,908	41,434	117
SW1	SAMD00571329	546,463	349,792	99,708	99,705	74,459	1,555
SW2	SAMD00571330	579,452	374,120	99,626	99,625	75,718	1,737
SW3	SAMD00571331	459,726	297,016	99,998	99,996	76,953	1,796
SW4	SAMD00571332	521,323	333,407	99,782	99,781	74,416	1,418
SW5	SAMD00571333	383,322	243,344	99,926	99,925	70,283	1,312
SS1	SAMD00571334	1,675,274	1,107,348	99,778	99,778	48,554	274
SS2	SAMD00571335	2,504,996	1,688,863	99,956	99,956	52,061	380
RW1	SAMD00571336	476,937	309,016	99,915	99,914	67,352	1,348
RW2	SAMD00571337	1,310,983	609,668	100,000	44,312	30,803	190

b Summary of 16S amplicon sequencing using an Illumina NovaSeq 6000 sequencer

ID	Environment	Accession No. (BioSample ID)	Number of reads (illumina)
SO1	Soil	SAMD00571322	1,269,207
SO2	Soil	SAMD00567467	1,090,457
SO3	Soil	SAMD00571323	1,177,853
SO4	Soil	SAMD00571324	1,387,164
SO5	Soil	SAMD00571325	1,228,252
SO6	Soil	SAMD00571326	1,001,786
SW1	Seawater	SAMD00571329	1,811,936
SW2	Seawater	SAMD00571330	1,937,300
SW3	Seawater	SAMD00571331	997,029
SW4	Seawater	SAMD00571332	1,853,564
SW5	Seawater	SAMD00571333	2,407,794
SS1	Seabed soil	SAMD00571334	1,807,534
SS2	Seabed soil	SAMD00571335	1,817,894
RW1	River water	SAMD00571336	2,557,360
RW2	River water	SAMD00571337	2,557,360

Supplementary Table S7. Percentage of "latent environment topics" in each environment sample collected in this study

Topic # and terms that descrive each topic	TW1	TW2	RW1	RW2	SO2	SO3	SO4	SO5	SO6	SS1	SS2	SW1	SW2	SW3	SW4	SW5
Topic0 (marine, mat, hydrothermal, vent, water)	-	-	0.9	0.4	-	-	-	-	-	-	2.2	0.5	0.7	1.1	-	1.3
Topic1 (marsh, marine, anthropogenic, fecal, sippewissett)	-	-	1.1	-	0.1	-	-	-	-	-	-	0.2	0.8	-	8.6	0.6
Topic2 (gut, ant, patient, marsh, fungus)	-	-	-	0.4	0.2	2.6	0.1	0.3	0.2	-	-	-	-	-	-	-
Topic3 (water, freshwater, baltic, lake, sea)	-	-	1.2	1.7	-	-	-	-	-	-	-	1.3	2.0	1.6	-	-
Topic4 (mosquito, acinetobacter, gambiae, anopheles, male)	-	-	-	-	-	0.2	-	-	-	-	-	-	-	-	-	-
Topic5 (root, endophyte, rhizosphere, compartment, germany)	-	-	0.7	1.8	1.1	-	-	-	-	-	-	-	-	-	-	-
Topic6 (soil, rhizosphere, field, garden, ant)	-	0.6	-	-	-	-	-	-	-	-	-	-	-	-	-	-
Topic7 (water, marine, sea, seawater, compartment)	-	-	-	-	-	-	-	-	-	-	-	-	0.4	0.2	-	-
Topic8 (hydrocarbon, tail, water, pond, oil)	-	-	0.4	-	-	-	-	-	-	-	-	-	-	-	-	-
Topic9 (gut, healthy, kwashiorkor, twin, fecal)	-	-	-	-	-	-	-	-	-	-	-	-	-	-	-	-
Topic10 (concrete, biofilms, corrosion, biofilm, rocky)	-	-	-	-	-	-	-	-	-	-	-	-	-	-	-	-
Topic11 (beach, sand, marine, surface, oil)	**27.4**	**40.8**	-	-	-	-	0.2	-	-	**20.1**	**30.2**	**24.3**	**27.9**	**23.9**	**38.9**	**20.4**
Topic12 (tonsil, diseased, oral, canal, root)	-	-	-	-	-	-	-	-	-	-	-	-	-	-	-	-
Topic13 (male, disease, naris, skin, female)	-	-	-	-	-	-	-	-	-	-	-	-	-	-	-	-
Topic14 (coral, skin, reef, marine, healthy)	-	-	-	-	-	-	-	-	-	0.3	-	-	-	-	-	-
Topic15 (dorsum, female, male, oral, plaque)	-	-	-	-	-	-	-	-	-	-	-	-	-	-	-	-
Topic16 (gut, mouse, fecal, gnotobiotic, kwashiorkor)	-	-	-	-	-	-	-	-	-	-	-	-	-	-	-	-
Topic17 (soil, forest, temperate, wood, peat)	-	-	-	0.2	**49.1**	**69.3**	**83.7**	**73.2**	**74.7**	-	0.1	0.5	0.6	0.6	0.4	0.4
Topic18 (soil, lettuce, sediment, arable, bovine)	-	-	-	-	-	-	-	-	-	-	-	-	0.4	0.7	-	0.2
Topic19 (soil, rhizosphere, plate, farm, root)	-	-	-	0.6	19.0	9.1	2.7	-	0.8	-	-	-	-	-	-	0.7
Topic20 (oral, female, saliva, vaginal, male)	-	-	0.2	-	-	-	-	-	-	-	-	-	-	-	-	-
Topic21 (esophageal, mucosa, adenocarcinoma, male, female)	-	-	0.1	-	-	-	-	-	-	-	-	-	-	-	-	-
Topic22 (mouse, fecal, diet, healthy, gnotobiotic)	-	-	-	-	-	-	-	-	-	-	-	-	-	-	-	-
Topic23 (crease, male, disease, skin, fossa)	-	-	-	-	-	-	-	-	-	-	-	-	-	-	-	-
Topic24 (gut, swine, feces, rumen, pig)	-	-	-	-	-	-	-	-	-	-	-	-	-	-	-	-
Topic25 (vaginal, gut, female, america, healthy)	-	-	-	-	-	-	-	-	-	-	-	-	-	-	-	-
Topic26 (spring, hot, sediment, archaeal, canada)	-	-	-	-	-	-	-	-	-	-	-	-	-	-	-	-
Topic27 (skin, forearm, volar, surface, hand)	-	-	-	-	-	-	-	-	-	-	-	-	-	-	-	-
Topic28 (gut, america, fecal, abdominal, recurrent)	-	-	-	-	-	-	-	-	-	-	-	-	-	-	-	-
Topic29 (dental, terrestrial, oral, biofilm, industrial)	-	-	5.4	0.2	-	-	-	-	-	-	-	0.2	-	-	-	0.1
Topic30 (male, female, tonsil, palate, dorsum)	-	-	-	-	-	-	-	-	-	-	-	-	-	-	-	-
Topic31 (gut, fecal, male, disease, feces)	-	-	0.2	-	-	-	-	-	-	-	-	-	-	-	-	-
Topic32 (female, male, vaginal, sapiens, perianal)	-	-	-	0.1	-	-	-	-	-	-	-	-	-	-	-	-
Topic33 (gut, stool, neonatal, sapiens, healthy)	-	-	-	0.1	-	0.8	-	-	-	-	-	-	-	-	-	-
Topic34 (sediment, marine, coastal, marsh, mat)	-	-	-	-	-	0.2	0.2	-	0.2	**30.0**	**31.3**	2.0	4.3	4.7	0.3	4.6
Topic35 (ant, mosquito, attine, gamboa, nest)	-	-	0.4	0.2	-	1.0	-	-	-	-	-	1.2	-	1.5	0.5	0.3
Topic36 (gut, terrestrial, aries, ovis, rumen)	-	-	-	-	4.8	0.8	3.9	-	-	-	-	0.1	-	-	-	-
Topic37 (female, vaginal, introitus, vagina, fornix)	-	-	-	-	-	0.4	-	-	-	-	-	-	-	-	-	-
Topic38 (disease, skin, hand, naris, male)	-	-	0.1	-	-	-	-	-	-	-	-	-	-	-	-	-
Topic39 (marine, minimum, oxygen, zone, ocean)	-	-	-	-	-	-	-	0.1	-	0.9	1.5	4.0	4.6	3.7	1.4	0.8
Topic40 (water, sea, marine, saline, ocean)	-	3.0	-	-	0.1	-	-	-	-	-	-	3.6	5.1	2.1	11.2	**24.0**
Topic41 (mouse, gut, diet, stool, feces)	-	-	-	-	-	-	-	-	-	-	-	-	-	-	-	-
Topic42 (horse, rumen, kingdom, gut, colon)	-	-	-	-	-	-	-	-	-	-	-	-	-	-	-	-
Topic43 (female, vaginal, introitus, fornix, scot)	-	-	-	-	-	-	-	-	-	-	-	-	-	-	-	-

(Legend on next page)

Topic44 (sand, oil, depth, normal, hydrocarbon)	-	0.2	0.7	-	-	0.8	-	-	-	-	-	1.1	1.4	1.5	-	0.1
Topic45 (america, arable, dog, organismal, door)	0.9	0.5	-	-	-	-	-	-	-	-	-	-	-	-	0.1	0.8
Topic46 (biofilm, water, drink, copper, grow)	**37.4**	10.7	0.3	1.7	1.7	1.2	-	-	-	-	-	-	0.1	-	-	-
Topic47 (mouse, fecal, gnotobiotic, recipient, kwashiorkor)	-	-	-	-	-	-	-	-	-	-	-	-	-	-	-	-
Topic48 (america, skin, dog, organismal, pet)	-	-	-	-	-	-	-	-	-	-	-	-	-	-	-	-
Topic49 (plaque, male, female, dental, kingdom)	-	-	-	-	-	-	-	-	0.9	-	-	-	-	-	-	-
Topic50 (gut, varians, ant, cephalotes, insect)	-	-	2.4	1.7	0.1	0.2	-	-	-	-	-	-	-	-	-	-
Topic51 (feces, gut, intestinal, pig, antibiotic)	-	-	0.2	-	-	-	-	-	-	-	-	-	-	-	-	-
Topic52 (vaginal, female, american, african, introitus)	-	-	-	-	-	-	-	-	-	-	-	-	-	-	-	-
Topic53 (water, freshwater, river, wastewater, lake)	-	-	**78.7**	**81.2**	-	-	0.5	0.2	0.3	-	-	4.2	2.5	5.0	2.1	1.8
Topic54 (soil, field, terrestrial, compost, agricultural)	-	-	0.7	1.8	**21.0**	9.2	7.5	**22.1**	**21.4**	**20.1**	**20.4**	2.6	4.3	4.2	0.9	1.5
Topic55 (spring, sediment, hot, anaerobic, canada)	0.9	1.6	0.5	5.2	0.7	0.6	-	2.1	0.9	17.0	7.9	0.8	1.6	1.2	-	1.5
Topic56 (sludge, activate, wastewater, bioreactor, plant)	9.1	10.0	2.5	0.6	0.8	0.4	-	-	0.2	-	-	0.7	0.4	-	0.3	0.2
Topic57 (sand, marine, beach, surface, fish)	-	-	-	-	-	-	-	-	-	-	-	-	-	-	-	-
Topic58 (marine, spring, lake, water, hot)	-	-	-	-	-	-	-	-	-	-	-	-	-	-	-	-
Topic59 (skin, sediment, disease, female, oil)	-	-	0.3	0.4	0.4	1.4	0.2	0.2	0.2	-	-	-	-	-	-	-
Topic60 (skin, healthy, canine, esophageal, milk)	0.3	-	-	-	-	-	-	-	-	-	-	-	-	-	-	-
Topic61 (esophageal, adenocarcinoma, stomach, foregut, adult)	-	-	-	-	-	-	-	-	-	-	-	0.2	-	-	-	-
Topic62 (male, gingiva, mucosal, mucosa, bovine)	-	-	-	-	-	-	-	-	-	-	-	-	-	-	-	-
Topic63 (marine, water, coastal, sea, seawater)	**20.6**	**28.5**	-	0.2	-	0.4	-	-	-	-	-	**48.0**	**36.8**	**40.9**	**34.1**	**39.9**
Topic64 (gut, feces, china, dietary, faecal)	-	-	0.2	-	-	-	-	-	-	-	-	-	-	-	-	-
Topic65 (phyllosphere, sativa, field, lettuce, vancomycin)	-	-	0.3	0.2	-	0.1	-	-	-	-	-	-	-	-	-	-
Topic66 (disease, skin, fossa, male, crease)	-	-	-	-	-	-	-	0.1	0.1	-	-	-	-	-	-	-
Topic67 (mouse, gut, mycobacterium, tuberculosis, stool)	-	-	-	-	-	-	-	-	-	-	-	-	-	-	-	-
Topic68 (gut, infant, pig, role, uncultivated)	-	-	1.4	0.5	-	-	0.2	-	-	-	0.6	0.2	0.4	0.4	0.1	0.3
Topic69 (bee, gut, sediment, marine, colony)	-	-	-	-	-	-	-	-	-	-	-	-	-	-	-	-
Topic70 (marine, sediment, sponge, sea, deep)	3.2	3.9	-	-	0.5	0.4	-	1.2	-	11.4	5.8	3.4	5.1	6.1	-	-
Topic71 (mine, tail, compost, spring, natural)	-	-	-	-	-	-	-	-	-	-	-	-	-	-	-	-
Topic72 (oral, healthy, dog, america, adult)	-	-	-	-	-	-	-	-	-	-	-	-	-	-	-	-
Topic73 (mouse, gut, fecal, gnotobiotic, healthy)	-	-	-	-	-	-	0.1	-	-	-	-	-	-	-	-	-
Topic74 (male, gingiva, female, mucosa, dorsum)	-	-	-	-	-	-	-	-	-	-	-	-	-	-	-	-
Topic75 (gut, mouse, musculus, mu, fermentation)	-	-	-	-	-	-	-	-	-	-	-	-	-	-	-	-
Topic76 (phyllosphere, field, lettuce, sativa, material)	-	-	0.7	0.4	0.1	0.7	-	-	-	-	-	0.6	0.5	0.3	0.8	0.4
Topic77 (sediment, digester, pit, anaerobic, mud)	-	-	-	-	-	0.1	0.2	-	-	-	-	-	-	-	-	-
Topic78 (kwashiorkor, gut, fecal, mouse, gnotobiotic)	-	-	-	-	-	-	-	-	-	-	-	-	-	-	-	-
Topic79 (gut, male, female, feces, stool)	-	-	-	-	-	-	-	-	-	-	-	-	-	-	-	-

In the Latent Environment Allocation (LEA) analysis, each environment was defined in terms of 80 topics (a group of terms representing different environments, such as marine, mat, or water). The table shows the percentage composition of the topics in each environmental sample. "–" indicates that the percentage of the topic was <0.1%. When the percentage was ≥20%, the number is shown in bold.

Supplementary Table S7. (continued)

Supplementary Table S8. Percentages of radiation-resistant bacteria and bacteria found in nuclear facilities in each environment

Types	Genus	TW1	TW2	SW1	SW2	SW3	SW4	SW5	RW1	RW2	SS1	SS2	SO2	SO3	SO4	SO5	SO6	Ref.
Radioresistant bacteria	*Acinetobacter*	-	-	-	-	-	-	-	0.1	0.2	-	0.2	-	-	-	-	-	[1]
	Deinococcus	-	-	-	-	-	-	-	-	-	-	-	-	-	-	-	-	[1]
	Hymenobacter	-	-	-	-	-	-	-	-	-	-	-	-	-	-	-	-	[1]
	Rubrobacter	-	-	-	-	-	-	-	-	-	-	-	-	-	-	-	-	[1]
	Methylobacterium	0.2	0.2	-	-	-	-	-	-	-	-	-	-	-	-	-	-	[1]
	Massilia	-	-	-	-	-	-	0.1	0.4	0.2	-	-	-	0.3	-	-	-	[2]
	Brevundimonas	-	-	-	-	-	-	-	0.2	-	-	-	-	-	-	-	-	[3]
Bacteria identified from nuclear fuel pools	*Limnobacter*	41.6	10.0	-	-	-	-	-	-	0.1	0.4	-	-	-	-	-	-	[4]
	Sphingopyxis	1.6	2.6	-	-	-	-	-	-	-	-	-	-	-	-	-	-	[5]
	Novosphingobium	1.1	0.9	0.1	-	-	-	-	1.2	0.8	0.2	0.6	-	-	-	-	-	[5,6]
	Methylotenera	1.4	0.3	-	-	-	-	-	0.5	0.3	-	-	-	-	-	-	-	[6,7]
	Methylobacterium	0.2	0.2	-	-	-	-	-	-	-	-	-	-	-	-	-	-	[8,9]
	Pseudonocardia	0.1	0.6	-	-	-	-	-	-	-	-	-	-	-	-	-	-	[10]

References:

1. Cox, M. M. & Battista, J. R. Deinococcus radiodurans — the consummate survivor. *Nature Reviews Microbiology* **3**, 882–892 (2005).
2. Cheptsov, V. *et al.* Survivability of Soil and Permafrost Microbial Communities after Irradiation with Accelerated Electrons under Simulated Martian and Open Space Conditions. *Geosciences* **8**, 298 (2018).
3. Dartnell, L. R., Hunter, S. J., Lovell, K. V., Coates, A. J. & Ward, J. M. Low-Temperature Ionizing Radiation Resistance of Deinococcus radiodurans and Antarctic Dry Valley Bacteria. *https://home.liebertpub.com/ast* **10**, 717–732 (2010).
4. Hayoun, K. *et al.* Proteotyping Environmental Microorganisms by Phylopeptidomics: Case Study Screening Water from a Radioactive Material Storage Pool. *Microorganisms* **8**, 1525 (2020).
5. Silva, R. *et al.* Microbial enrichment and gene functional categories revealed on the walls of a spent fuel pool of a nuclear power plant. *PLoS One* **13**, e0205228 (2018).
6. Ruiz-Lopez, S. *et al.* Identification of a Stable Hydrogen-Driven Microbiome in a Highly Radioactive Storage Facility on the Sellafield Site. *Front Microbiol* **11**, 587556 (2020).
7. MeGraw, V. E. *et al.* A novel adaptation mechanism underpinning algal colonization of a nuclear fuel storage pond. *mBio* **9**, 10-1128 (2018).
8. Chicote, E. *et al.* Isolation and identification of bacteria from spent nuclear fuel pools. *J Ind Microbiol Biotechnol* **32**, 155–162 (2005).
9. Bagwell, C. E., Noble, P. A., Milliken, C. E., Li, D. & Kaplan, D. I. Amplicon sequencing reveals microbiological signatures in spent nuclear fuel storage basins. *Front Microbiol* **9**, 333128 (2018).
10. Bruhn, D. F., Frank, S. M., Roberto, F. F., Pinhero, P. J. & Johnson, S. G. Microbial biofilm growth on irradiated, spent nuclear fuel cladding. *Journal of Nuclear Materials* **384**, 140–145 (2009).

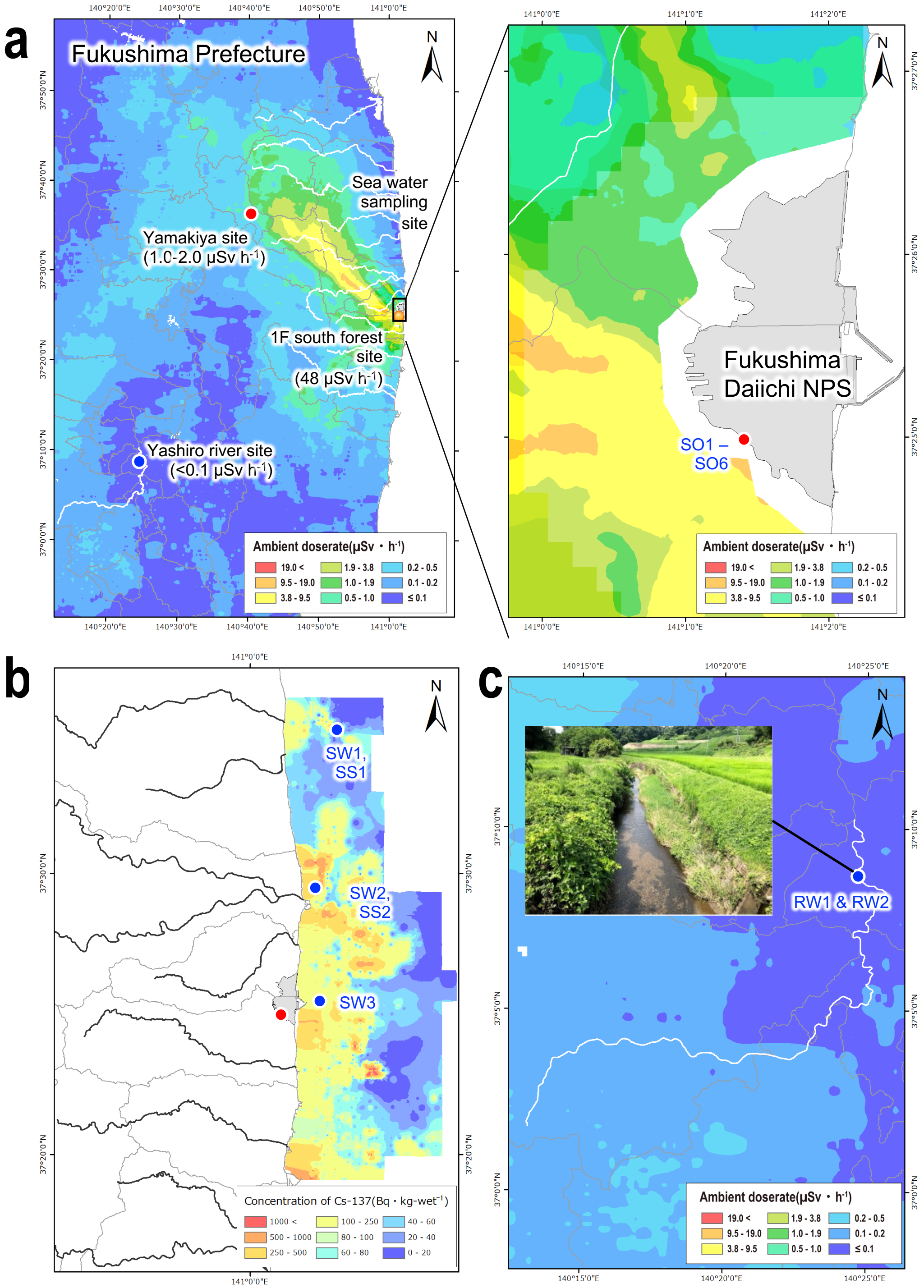

Supplementary Fig. S1. Collection sites of Fukushima environmental samples analyzed in this study. (a) Location of Fukushima Daiichi Nuclear Power Station (NPS) in Fukushima Prefecture, Japan (left) and enlarged view of the Fukushima Daiichi NPS region (right). Locations at which soil and water environmental samples were collected are indicated by red and blue circles, respectively. For samples from the Yamakiya area, see the paper by Hoyos-Hernandez *et al.* (Ref. 1). (b) Collection sites of marine samples off the coast of Fukushima and radioactive cesium concentrations. (c) Location and photograph of Yashiro River in Fukushima Prefecture, where freshwater samples were collected. Results of the radiation survey conducted by the Regulatory Agency in 2019 with a manned helicopter and an unmanned helicopter (JAEA, Database for Radioactive Substance Monitoring Data, https://emdb.jaea.go.jp/emdb/top) are used as the background figures for (a) and (c). Results of a ship-towed radiation survey conducted by the Regulatory Agency in 2014 (Ref. 2) are used as the background figure for (b). See Supplementary Table 1 for each environmental sample.

References:

1. Hoyos-Hernandez, C. *et al.* Community structure and functional genes in radionuclide contaminated soils in Chernobyl and Fukushima. *FEMS Microbiol Lett* **366**, 180 (2019).
2. Sanada, Y. *et al.* Visualization of radiocesium distribution in surface layer of seafloor around Fukushima Daiichi Nuclear Power Plant. *Scientific Reports* **11**, 1–13 (2021).

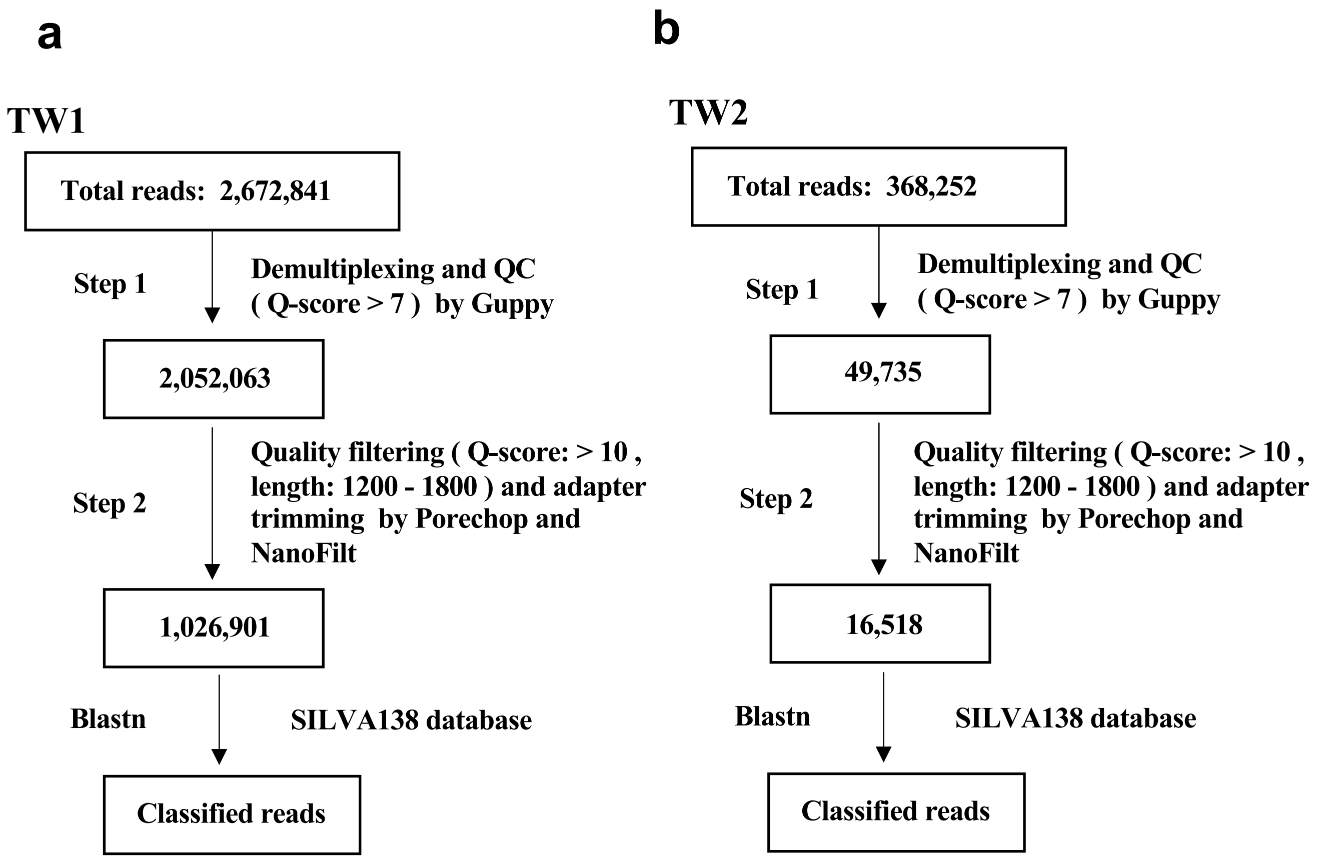

Supplementary Fig. S2. Sequence analysis pipeline with the MinION sequencer. Two pipelines for the analysis of nucleotide sequence data obtained from torus room waters (a) TW1 and (b) TW2. In Step 1, the quality of the nucleotide sequence was checked using Guppy. In Step 2, Porechop and NanoFit were used for adapter removal, quality filtering, and read selection by sequence length. The resulting sequences were classified with a sequence similarity search of the Silva 138 database with BLASTN. Numbers inside the boxes indicate the numbers of reads that passed each filtering step. See Supplementary Table S6a for "Classified reads."

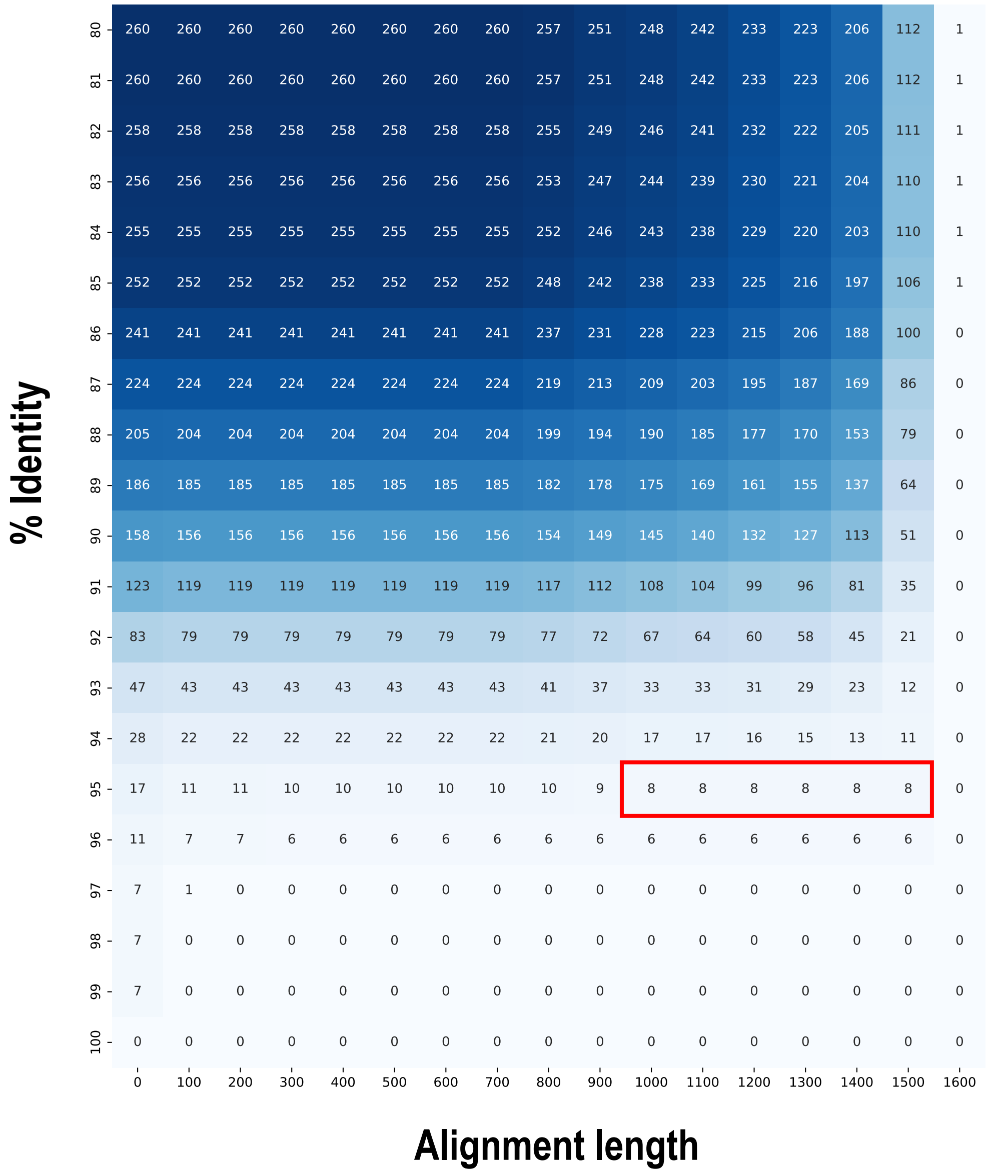

Supplementary Fig. S3. Relationship between BLAST alignment length and percentage identity using bacterial mock communities. After the mock bacterial community (D6300, Zymo Research, USA) was analyzed in the same way as the torus room water samples, the nucleotide sequence was determined. The results of a sequence similarity search of the Silva 138 database with BLASTN are shown in a heatmap of percentage identity on BLASTN on the vertical axis and alignment length on the horizontal axis. The mock community was a mixture of eight species (genera) of bacteria and two species of yeast. Numbers on the heatmap indicate the numbers of genera identified. The red box indicates the region corresponding to eight bacterial genera that correctly classified the mock community.

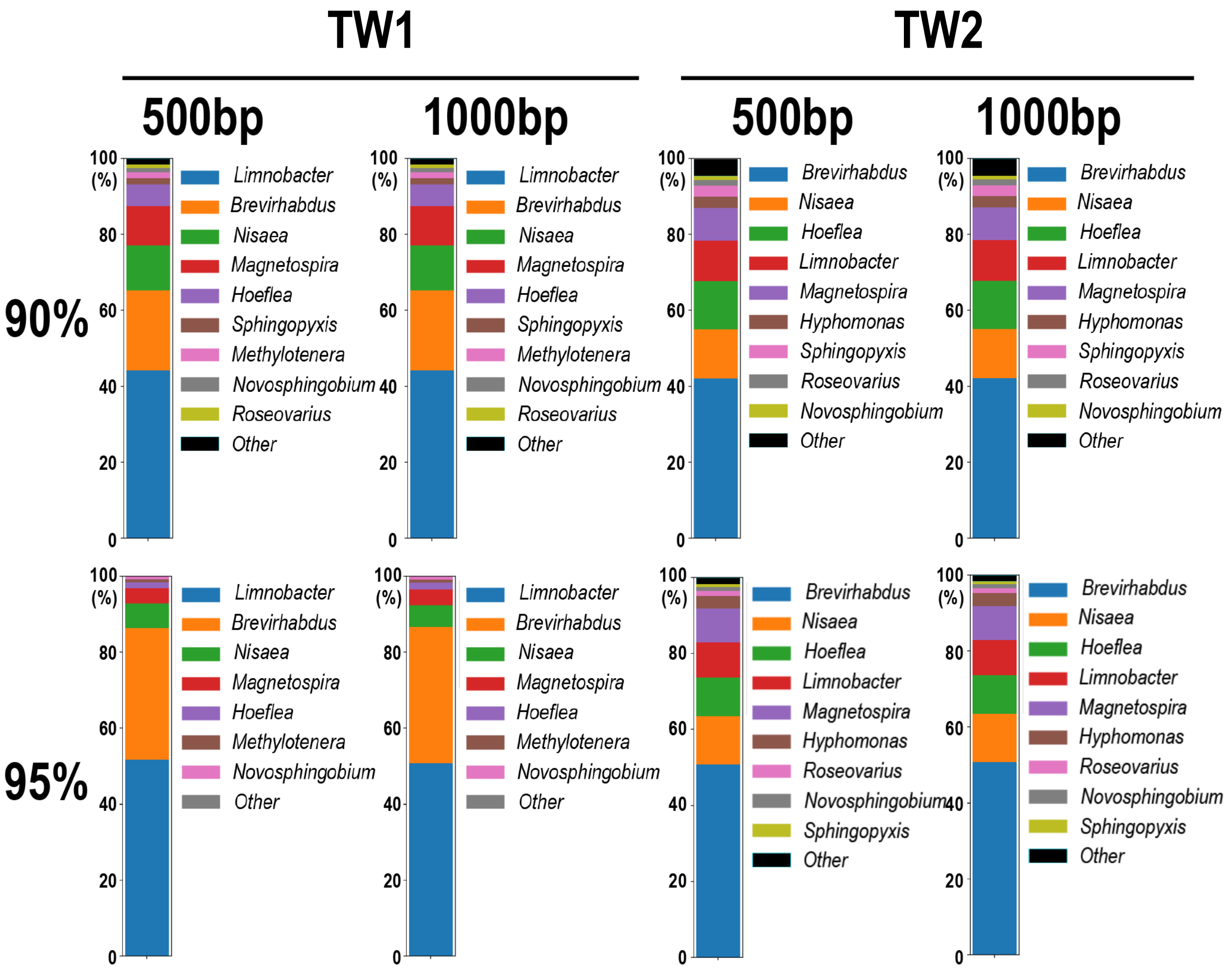

Supplementary Fig. S4. Changes in microbial community structure derived from torus room waters due to differences in BLAST alignment length and percentage identity. To estimate the microbial community structures in the torus room waters, each full-length 16S rRNA gene was amplified and sequenced using the MinION sequencer. To classify the sequences, a sequence similarity search with BLASTN was performed against the Silva 138 database for two alignment lengths (>500 bp or >1000 bp) and two degrees of similarity (>90% or >95%). The search results are shown as each microbial community structure at the genus level.

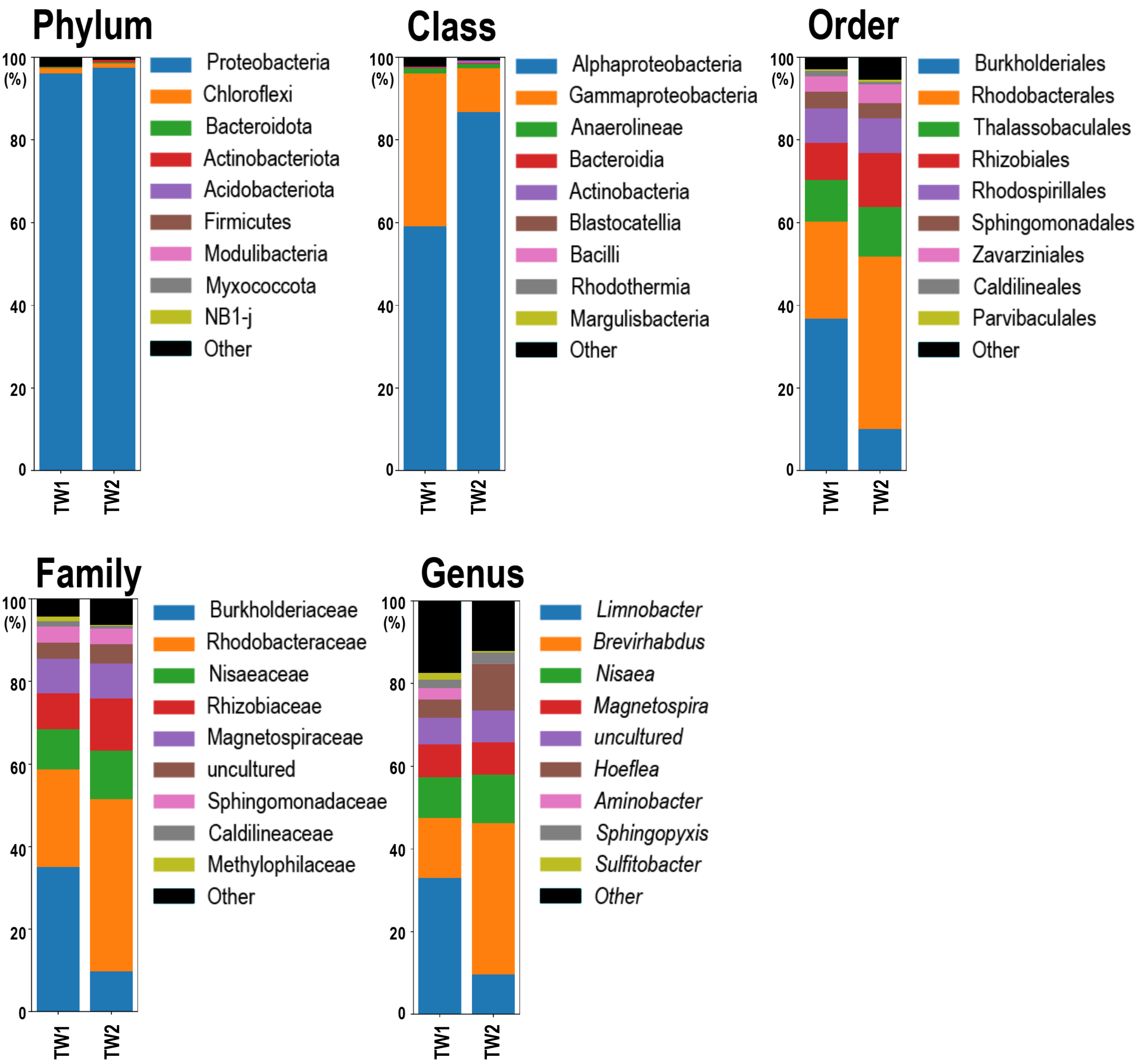

Supplementary Fig. S5. Microbial community structures in torus room waters at each taxon level. The microbial community structures in torus room waters TW1 and TW2 are shown at five levels: kingdom, phylum, class, order, family, and genus. Nucleotide sequences of full-length 16S rRNAs in each environmental sample were determined with the Oxford Nanopore Technologies MinION sequencer. To estimate the microbial community structures in the torus room waters, each full-length 16S rRNA gene was amplified and sequenced with the MinION sequencer. To classify the sequences, a sequence similarity search with BLASTN was performed in the Silva 138 database, with a minimum alignment length of 1000 bases and a minimum identity of 90%.

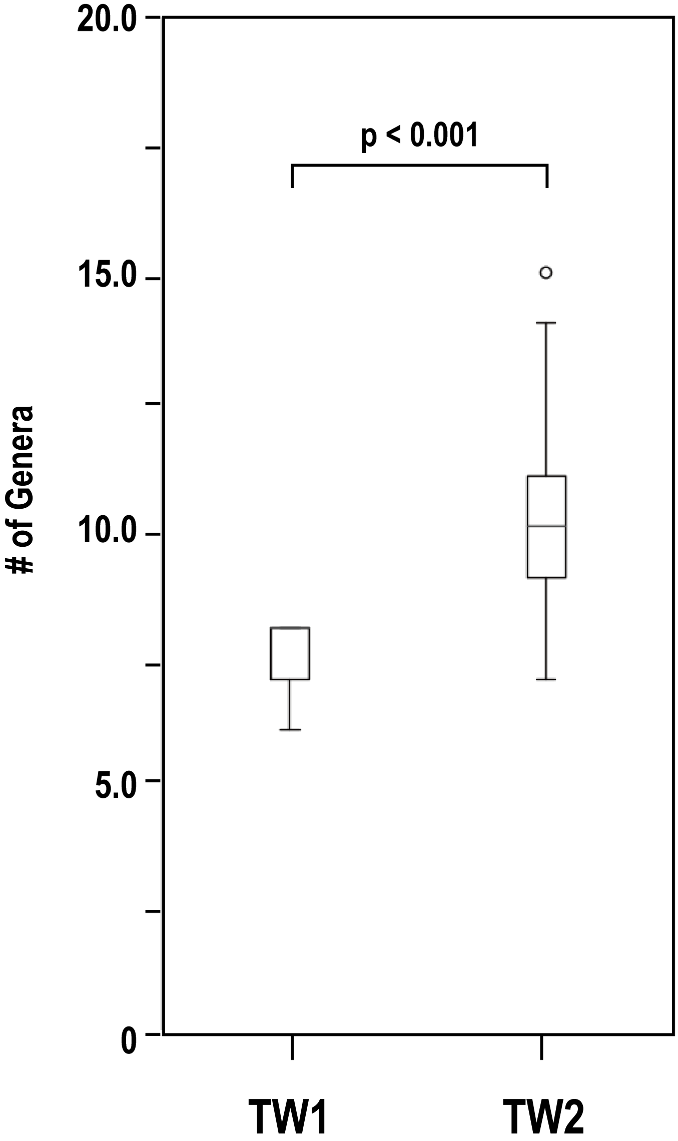

Supplementary Fig. S6. Comparison of numbers of genera identified in torus room water samples TW1 and TW2. Box plots show the number of genera identified by randomly extracting 100 reads from the 16S rRNA sequence reads obtained from each torus room water sample. Random extraction was performed 50 times. The circles in the figure indicate an outlier value. The p value was determined using Welch's *t* test.

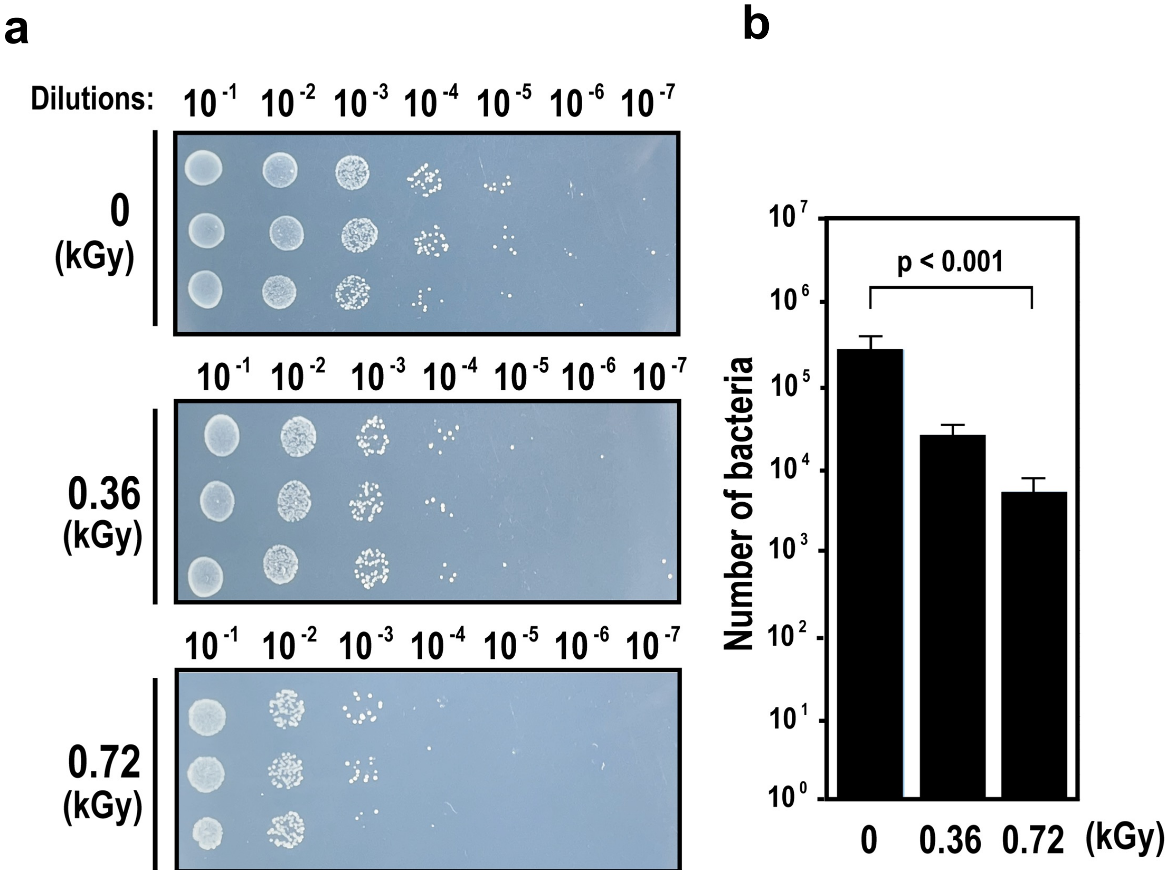

Supplementary Fig. S7. γ-Irradiation of *Limnobacter thiooxidans* CS-K2. (a) A liquid culture of *L. thiooxidans* was γ-irradiated at 0, 2.5, or 5 Gy/h for 6 days, then serially diluted and cultured on agar plates for 48 h (see Materials and Methods section for details). The photographs show the plates under each level of irradiation (n = 3). (b) Changes in γ-irradiation intensity and numbers of viable bacteria. Error bars indicate standard deviations, and p values were determined using a *t* test.

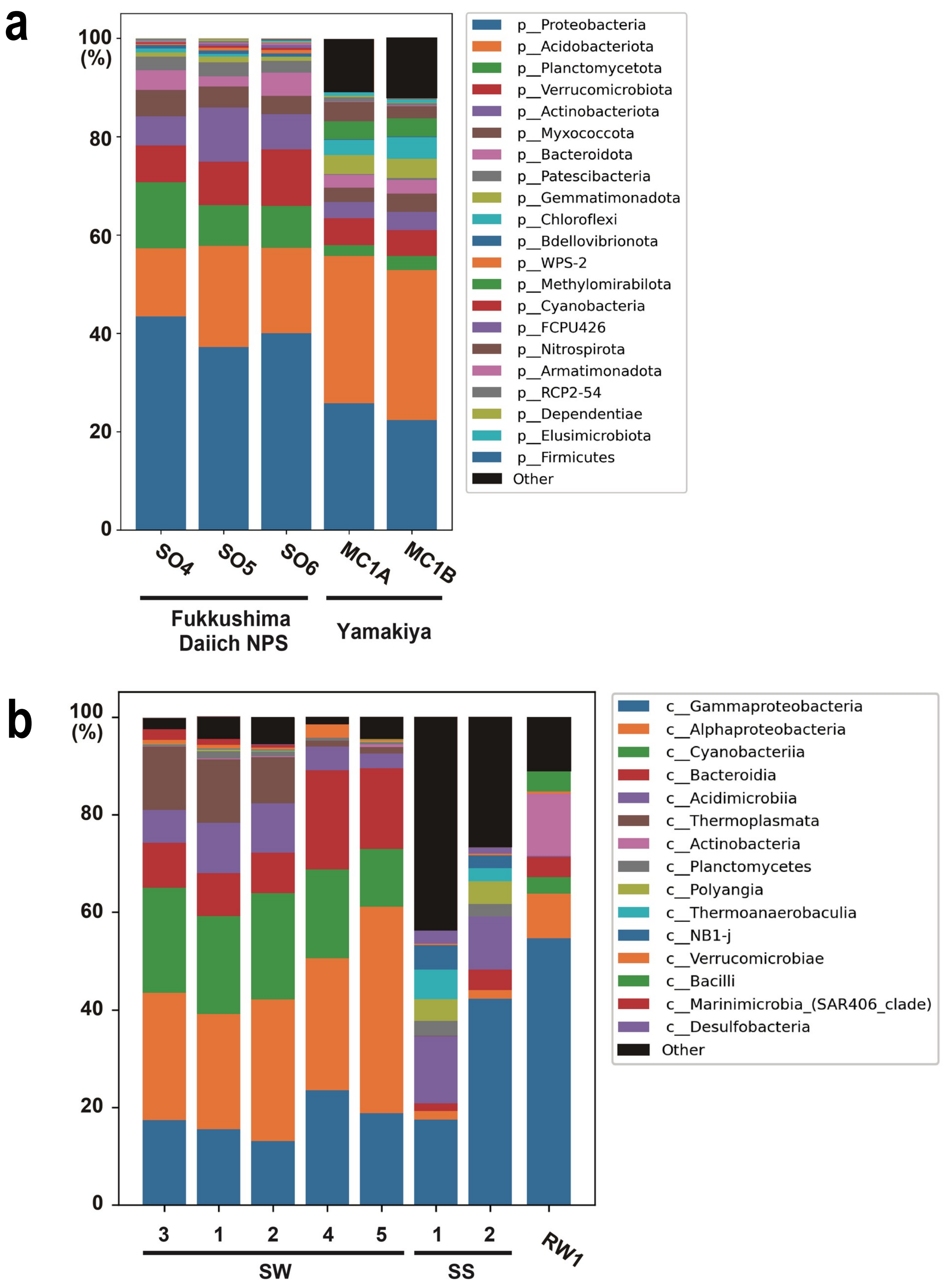

Supplementary Fig. S8. Analysis of microbial community structures in Fukushima environmental samples with an Illumina sequencer. (a) Comparison of microbial community structures (at the phylum level) in soil samples from Fukushima Daiichi NSP South Forest and soil samples from the Yamakiya area. (b) Comparison of microbial community structures (at class level) among Fukushima water samples. The V3–V4 region of the 16S rRNA gene was amplified from genomic DNA samples collected from each environment and sequenced using an Illumina sequencer. The microbial community structures were estimated based on the nucleotide sequences determined. See Supplementary Table 1 for each environmental sample.

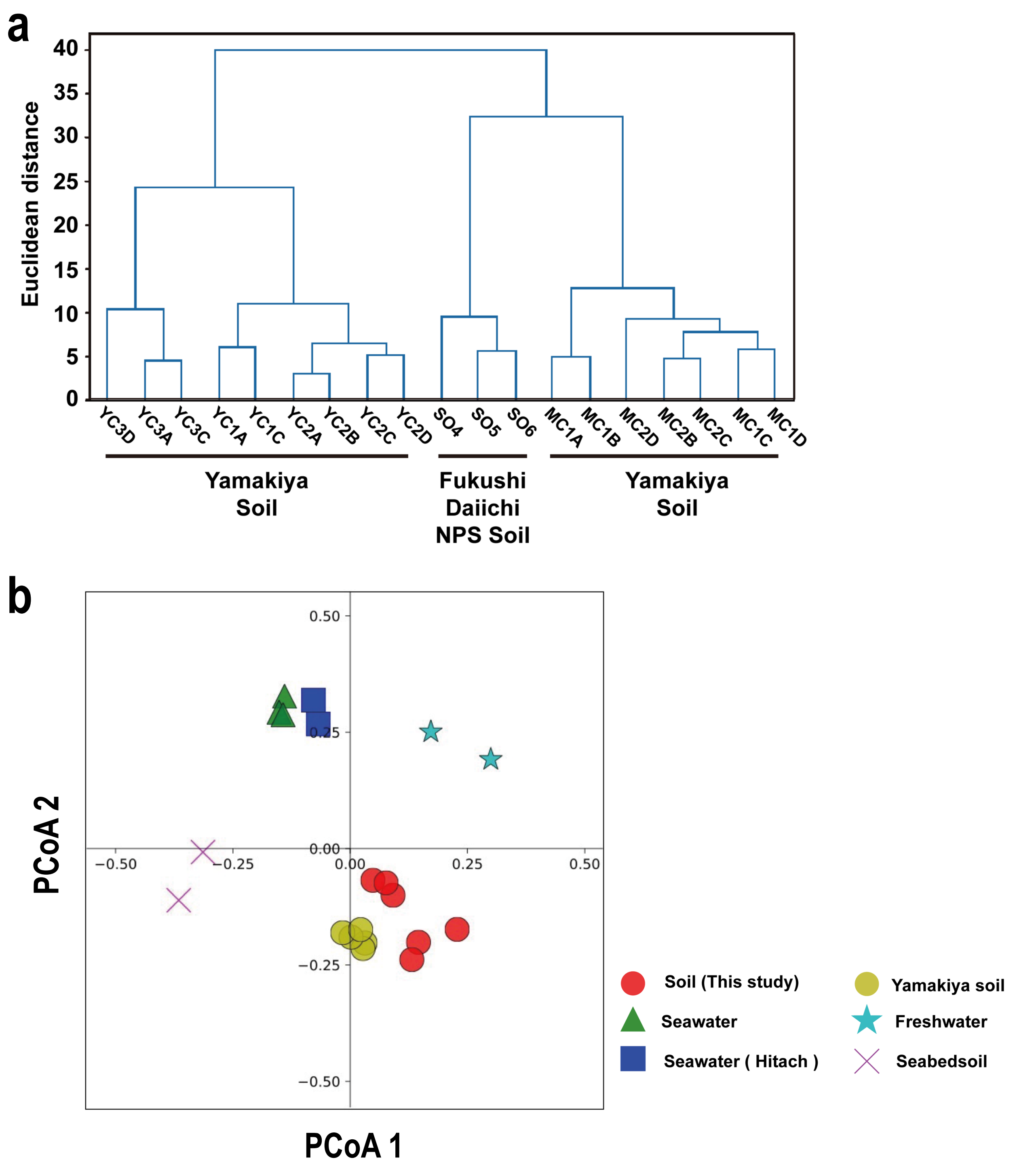

Supplementary Fig. S9. Comparison of microbial community structures in Fukushima environmental samples. (a) Hierarchical clustering analysis of microbial community structures near Fukushima Daiichi NPS. The V3–V4 region of the 16S rRNA gene was amplified from genomic DNA samples collected from each environment and sequenced using an Illumina sequencer. The microbial community structures were estimated based on the nucleotide sequences determined. A hierarchical clustering analysis of class-level microbial community profiles was performed with Ward's method. (b) Principal coordinates analysis (PCoA) plot of environmental samples collected in this study. Nucleotide sequences of 16S rRNA were determined using the method described in Supplementary Fig. S8a, and the PCoA plot was constructed from class-level microbial community profiles.

		TW 1	TW 2	SNFP	RW 1	RW 2	SO 2	SO 3	SO 4	SO 5	SO 6	SS 1	SS 2	SW 1	SW 2	SW 3	SW 4	SW 5
TW	1	1.00	1.00	0.50	0.38	0.12	0.25	0.25	0.12	0.12	0.12	0.12	0.12	0.12	0.12	0.12	0.12	0.12
	2	0.24	1.00	0.33	0.18	0.03	0.09	0.12	0.03	0.03	0.03	0.03	0.03	0.06	0.06	0.06	0.06	0.09
SNFP		0.04	0.12	1.00	0.24	0.09	0.14	0.11	0.03	0.01	0.02	0.01	0.00	0.02	0.02	0.03	0.02	0.04
RW	1	0.03	0.06	0.23	1.00	0.25	0.12	0.14	0.02	0.02	0.01	0.03	0.03	0.09	0.07	0.08	0.08	0.19
	2	0.02	0.02	0.20	0.59	1.00	0.22	0.15	0.05	0.05	0.02	0.02	0.05	0.10	0.12	0.10	0.15	0.29
SO	2	0.03	0.04	0.19	0.16	0.13	1.00	0.42	0.23	0.16	0.14	0.03	0.04	0.12	0.13	0.14	0.13	0.13
	3	0.04	0.07	0.18	0.23	0.11	0.51	1.00	0.26	0.18	0.18	0.04	0.05	0.05	0.05	0.12	0.05	0.12
	4	0.06	0.06	0.18	0.12	0.12	0.94	0.88	1.00	0.53	0.47	0.06	0.12	0.12	0.12	0.24	0.12	0.18
	5	0.07	0.07	0.07	0.14	0.14	0.79	0.71	0.64	1.00	0.57	0.07	0.21	0.14	0.14	0.21	0.14	0.21
	6	0.08	0.08	0.17	0.08	0.08	0.83	0.83	0.67	0.67	1.00	0.08	0.17	0.08	0.08	0.25	0.08	0.17
SS	1	0.02	0.02	0.02	0.07	0.02	0.05	0.05	0.02	0.02	0.02	1.00	0.62	0.17	0.14	0.14	0.05	0.12
	2	0.02	0.02	0.00	0.06	0.04	0.06	0.06	0.04	0.06	0.04	0.55	1.00	0.28	0.19	0.23	0.06	0.21
SW	1	0.02	0.03	0.03	0.14	0.06	0.13	0.05	0.03	0.03	0.02	0.11	0.21	1.00	0.59	0.63	0.51	0.52
	2	0.02	0.04	0.04	0.12	0.09	0.16	0.05	0.04	0.04	0.02	0.11	0.16	0.66	1.00	0.64	0.55	0.54
	3	0.02	0.03	0.05	0.12	0.06	0.15	0.11	0.06	0.05	0.05	0.09	0.17	0.62	0.55	1.00	0.51	0.54
	4	0.02	0.04	0.04	0.15	0.12	0.17	0.06	0.04	0.04	0.02	0.04	0.06	0.62	0.60	0.63	1.00	0.63
	5	0.01	0.04	0.06	0.25	0.17	0.13	0.10	0.04	0.04	0.03	0.07	0.14	0.46	0.42	0.49	0.46	1.00

Supplementary Fig. S10. Numbers of commonly identified bacterial genera in each environmental sample and the rates of co-detection (supporting data for Fig. 4). Ratios of microbial genera identified in common between the environmental samples are shown on the vertical and horizontal axes. A higher ratio is indicated by a larger circle. See Supplementary Table S1 for each environmental sample.
